# Phytocompounds and Regulation of Flavonoids in In Vitro-Grown Safflower Plant Tissue by Abiotic Elicitor CdCl_2_

**DOI:** 10.3390/metabo14020127

**Published:** 2024-02-16

**Authors:** Bushra Ejaz, Abdul Mujib, Rukaya Syeed, Jyoti Mamgain, Moien Qadir Malik, Kanchan Birat, Yaser Hassan Dewir, Katalin Magyar-Tábori

**Affiliations:** 1 Cellular Differentiation and Molecular Genetics Section, Department of Botany, Jamia Hamdard, New Delhi 110062, India; bushraejaz_sch@jamiahamdard.ac.in (B.E.); rukayasyeed_sch@jamiahamdard.ac.in (R.S.); jyotimamgain_sch@jamiahamdard.ac.in (J.M.); mqmalik_sch@jamiahamdard.ac.in (M.Q.M.); kanchan_sch@jamiahamdard.ac.in (K.B.); 2 Plant Production Department, College of Food and Agriculture Sciences, King Saud University, Riyadh 11451, Saudi Arabia; ydewir@ksu.edu.sa; 3 Research Institute of Nyíregyháza, Institutes for Agricultural Research and Educational Farm (IAREF), University of Debrecen, P.O. Box 12, 4400 Nyíregyháza, Hungary; mtaborik@agr.unideb.hu

**Keywords:** *Carthamus tinctorius* L., elicitation, medicinal plants, phytochemicals, regeneration, somatic embryogenesis

## Abstract

In this study, a Gas chromatography–mass spectrometry (GC–MS) investigation of embryogenic callus and somatic embryo regenerated shoots of *Carthamus tinctorius* revealed the presence of a variety of sugars, sugar acids, sugar alcohols, fatty acids, organic acids, and amino acids of broad therapeutic value. The in vitro developed inflorescence contained a wide range of active compounds. In embryogenic calluses, important flavonoids like naringenin, myricetin, kaempferol, epicatechin gallate, rutin, pelargonidin, peonidin, and delphinidin were identified. To augment the synthesis of active compounds, the effect of cadmium chloride (CdCl_2_) elicitation was tested for various treatments (T1–T4) along with a control (T0). Varying concentrations of CdCl_2_ [0.05 mM (T1), 0.10 mM (T2), 0.15 mM (T3), and 0.20 mM (T4)] were added to the MS medium, and flavonoid accumulation was quantified through ultra-high-pressure liquid chromatography–tandem mass spectroscopy (UHPLC–MS/MS). The flavonoids naringenin, kaempferol, epicatechin gallate, pelargonidin, cyanidin, and delphinidin increased by 6.7-, 1.9-, 3.3-, 2.1-, 1.9-, and 4.4-fold, respectively, at T3, whereas quercetin, myricetin, rutin, and peonidin showed a linear increase with the increase in CdCl_2_ levels. The impacts of stress markers, i.e., ascorbate peroxidase (APX), catalase (CAT), and superoxide dismutase (SOD), on defense responses in triggering synthesis were also evaluated. The maximum APX and SOD activity was observed at T3, while CAT activity was at its maximum at T2. The impact of elicitor on biochemical attributes like protein, proline, sugar, and malondialdehyde (MDA) content was investigated. The maximum protein, proline, and sugar accumulation was noted at high elicitor dose T4, while the maximum MDA content was noted at T3. These elevated levels of biochemical parameters indicated stress in culture, and the amendment of CdCl_2_ in media thus could be a realistic approach for enhancing secondary metabolite synthesis in safflower.

## 1. Introduction

Medicinal plants play an important role in the production of a wide range of medicines, and hence, botanical products are becoming attractive to the nutraceutical and pharmaceutical industries over synthetic medicines. The increased demand for crude drugs encourages the cultivation of many medicinal plants, such as safflower (*Carthamus tinctorius* L.) [1]. Plant secondary metabolites comprise a diversity of low-molecular-weight compounds. These are engaged in plant defense against biotic and abiotic stressors, helping plants to cope with altered environmental conditions. In response to adverse situations, various complex signaling systems of plants are activated, resulting in the successful development of plant defense mechanisms.

Safflower is an annual herbaceous oilseed belonging to the Asteraceae family. It is a hyperaccumulator and can grow under a variety of heavy metal (Cd, Ni, Pb, Zn) stresses. It can accumulate and translocate metals to aerial parts, accentuating its phytoextraction potential [2]. In addition, safflower plant parts synthesize alkaloids, flavonoids, polyacetylene, aromatic glucosides, organic acids, and many bioactive compounds that demonstrate anti-inflammatory, antidiabetic, antitumor, neuroprotective, anti-oxidant, and immunostimulant activities [3]. Safflower research emphasizes the identification of important bioactive compounds present in various plant parts involved in plant growth and development and defense mechanisms.

Gas chromatography–mass spectrometry (GC–MS) is a frequently used technique in detecting phytocompounds present in in vitro-grown plant tissue [4,5]. This methodology is extensively utilized in chemistry, plant science, pharmacology, biotechnology, and biochemistry [6]. It first separates components from a mixture, identifies unknown compounds, and determines the chemical structure, molecular weight, and other properties [7]. GC–MS quantifies volatile organic compounds, plant growth regulators, secondary metabolites, and other metabolic compounds in several medicinal important plants like Madagascar Periwinkle (*Catharanthus roseus* L.) [8], rasna (*Pluchea lanceolata* (DC.) C.B. Clarke) [9], thyme (*Thymus vulgaris* L.), and basil (*Ocimum basilicum* L.) [10].

In recent years, several biotechnological strategies like media optimization with plant growth regulators, the use of high-yielding cells, the addition of precursors, the over-expression of key enzymes, and the incorporation of various elicitors have been used to enhance the accumulation of bioactive compounds [11,12]. The regulatory influence of diverse biotic and abiotic compounds as elicitors on secondary metabolite enrichment has also been studied [13,14]. Among abiotic compounds, heavy metals have been extensively employed in several plant species to enhance plant growth and development and in the accumulation of phytochemicals [15,16].

The identification of new compounds, i.e., the study of metabolomics, is valuable in molecular networking and fills the gaps between genotypes and phenotypes. It has a multidisciplinary role in gene discovery and taxonomic fingerprinting and in studying biochemical enzymatic reactions. It may also be used for the comparative analysis of agricultural cultivars, food products, and traditional medicine [17]. Owing to high metabolome coverage and versatility, liquid chromatography–mass spectrometry (LC–MS) and MS have been utilized and are considered to be valuable platforms for metabolomics research. Ultra-high-pressure liquid chromatography (UHPLC) has seen extensive applications in plant metabolomics in the last decade as it shows high peak capacity and separation efficiency [18]. LC–MS/MS has recently been utilized for the identification of bioactive compounds in in vivo and in vitro plant extracts in different medicinal plant genera like *Eugenia* [19] and *Saraca* [20].

It is known that in elicitor treatment, tissues show elevated levels of reactive oxygen species (ROS), which are signaling molecules with a role in modulating significant protein activities, gene expression, and metabolic fluxes [21]. The chloroplasts, mitochondria, peroxisomes, and apoplasts are the major ROS-producing sites during abiotic stress [22]. However, accumulated ROS, if left unchecked, may result in oxidative stress, causing damage to the DNA, RNA, and protein macromolecules and lipid membrane peroxidation. This oxidative burst is mitigated by many ROS-detoxifying proteins, which consist of enzymatic anti-oxidants, such as catalase (CAT), superoxide dismutase (SOD), and ascorbate peroxidase (APX), present in cellular sub-compartments, as well as non-enzymatic compounds like anthocyanins, flavonoids, and phenolics [23].

The aim of the present study was to identify bioactive compounds present in methanolic extracts of embryogenic callus, shoot, and inflorescence of tissue-culture-derived plantlets through GC–MS. In this study, CdCl_2_ (cadmium chloride) was used as an abiotic elicitor, and the yield of phytocompounds, especially different flavonoids, was measured in regenerated tissues through UHPLC–MS/MS quantitative analysis. The study identified diverse flavonoids like anthocyanidin, flavonols, flavanone, flavanol glycoside, and flavan-3-ols, which can serve as potential anti-oxidants in *C. tinctorius.* The changes in biochemical attributes like the content of sugar, protein, and proline and the activity of anti-oxidant enzymes in response to CdCl_2_ treatment were also monitored.

## 2. Materials and Methods

### 2.1. Establishment of In Vitro Culture

The basic protocol of in vitro seed germination of *Carthamus tinctorius* L. was established by following the method laid out by the authors in [24]. The seeds of the ISF-764 variety of safflower were provided by the Indian Council of Agricultural Research—Indian Institute of Oilseeds Research (ICAR—IIOR) during the harvesting season March–April (2021). Overnight pre-soaked safflower seeds were surface-sterilized by washing them under running water with detergent (cetrimide). This was followed by a series of seed-surface sterilizations under a laminar flow cabinet to ensure the inoculation of uncontaminated seeds. For this, safflower seeds were transferred in a glass beaker and soaked twice in 70% ethanol for 90 s and rinsed with autoclaved double-distilled water (DDW) thrice after each treatment. These ethanol-treated seeds were further stirred twice in 0.1% HgCl_2_ (*w*/*v*) by manually shaking the beaker for 90 s, followed by soaking (20 s) and rinsing with DDW thrice after each treatment to remove any traces of disinfectant. The treatment time was monitored using a stopwatch and has been optimized for safflower seeds used in this experiment.

The surface-sterilized seeds were inoculated on half-strength MS [25] supplemented with 1.4 μM GA_3_, 1.5 g/100 mL sucrose, and 0.7% agar. The pH of the germination medium was set at 5.7 and sterilized in an autoclave at 121 °C for 15 min. The culture tubes with inoculated seeds were incubated in dim light for 3–4 days until the appearance of hypocotyls. Later, the cultures were subject to a 16/8 h photoperiod regime under 100 μmol m^−2^ s^−1^ photon flux density (PPFD) provided by cool white fluorescent tube lights at 25 °C.

For the CdCl_2_ elicitation of flavonoids, the somatic embryo regenerated plantlets of *C. tinctorius* were cultured in MS basal medium with different treatments of CdCl_2_ (0.05, 0.10, 0.15, and 0.20 mM), represented as T1, T2, T3, and T4, respectively, and untreated versions (T0) were used as the control for comparative analysis. After one week of incubation, all the samples (T0, T1, T2, T3, and T4) were harvested and processed for metabolomic analysis.

### 2.2. Embryogenic Callus Induction and Shoot Regeneration

For callus formation, the in vitro-germinated 2-week-old seedlings were inoculated on MS supplemented with 2.2 µM BAP + 5.4 µM NAA + 2.27–4.54 µM TDZ. The embryogenic callus was obtained from a hypocotyl-derived non-embryogenic callus after eight weeks of subculturing in an MS medium. For germination, the somatic embryos were cultured in a varying-strength MS medium (F-MS and ½ MS) supplemented with GA_3_ (0.29–1.4 µM). Shoot buds obtained from somatic embryos were cultured on MS supplemented with 2.2 µM BAP and 4.54–9.08 µM TDZ along with 0.54 µM NAA for multiplication and elongation. The frequency (%) of embryogenesis, the mean number of somatic embryos per callus clump (100 mg), the germination frequency (%) of embryos, and the mean number of in vitro-regenerated shoots and flower buds per plantlet were recorded.

### 2.3. Metabolomic Profiling of Somatic Embryogenic Callus and Regenerated Shoot of Safflower: Extract Preparation and Derivatization for Gas Chromatography–Mass Spectrometry (GC–MS)

For untargeted GC–MS analysis, the standard Kundu et al. method was followed [26]. An extract of 480 µL of pure methanol along with 20 µL of 0.2 mg mL^−1^ ribitol (adonitol) as internal standard was added to 20 mg of dried callus and shoot sample. The mixture was vigorously shaken for 2 min and then heated at 70 °C for 15 min (ThermoStatC, Eppendorf, Hamburg, Germany). To this, an equal volume of water was added and vortexed (Spinix vortex shaker, Tarsons, Mumbai, India), followed by the addition of 250 µL of chloroform and thoroughly mixed. This mixture was centrifuged (Eppendorf ^R^ centrifuge 5430 R) at 2200× *g* for 10 min at room temperature (~22–25 °C). The upper aqueous phase was pipetted out and dried in a speed vacuum rotator (Concentrator plus, Eppendorf) at 45 °C for 2.5 h. The dried fraction was redissolved in 40 µL of 20 mg mL^−1^ methoxamine hydrochloride prepared in pyridine and then incubated for 90 min at 30 °C (ThermoStatC, Eppendorf). A total of 60 µL of MSTFA (N-methylN-(trimethylsilyl) trifluroacetamide) was added to the above solution and incubated for 30 min at 37 °C. A total of 100 µL of this derivatized sample was transferred in an insert containing a GC–MS glass vial and stored at 4 °C until it was analyzed in GC–MS/MS (TQ8050 NX, Shimadzu, Kyoto, Japan).

### 2.4. GC–MS Analysis and Data Processing

Following derivatization, the injection was set at Split Mode, with a split ratio of 5 and 0.2 μL injection volumes. The GC–MS analysis for untargeted metabolites consisted of a Shimadzu Gas chromatogram (GC-2010 plus) coupled with a mass spectrometer (TQ 8050), and an auto-sampler (AOC-20s) and auto-injector (AOC-20i) was used. For analysis, helium carrier gas (flow rate 1 mL min^−1^) and SH-Rxi-5Sil MS capillary column (30 m × 0.25 μm, 0.25 mm) (Restek Corporation, Bellefonte, PA, USA) were used. The system was set at 80 °C isothermal heating (2 min), followed by a ramp rate of 5 °C min^−1^ to 250 °C, 2 min withhold, and a 10 °C min^−1^ final ramp with 24 min withhold time. The total run time for GC–MS was 68 min with 4.5 min of solvent delay [27]. The GC–MS solution software Version 4.45 SP 1 was applied for chromatogram integration and analysis of mass spectra. The derivatized metabolites were identified using NIST14s and the WILEY8 spectral library.

### 2.5. Preparation of Inflorescence Methanolic Extract for GC–MS Analysis and Identification of Phytochemicals

The in vitro-derived capitulum was harvested and dried under sterilized conditions and finely ground using a mortar and pestle. This pulverized tissue was soaked in 100% methanol (1.0 gm in 5.0 mL) and stirred on a rotary shaker for 15 h. The extracts were filtered using a 0.22 m pore-size syringe filter for 10 min. The extracts were centrifuged at 8000 rpm and 4 °C. The resulting supernatant was then pipetted out (leaving the debris) into fresh GC–MS vials, and 1.0 µL of each sample was utilized for GC–MS analysis. The sample extract was delivered to the University Science Instrumentation Centre, AIRF, Jawaharlal Nehru University in Delhi for GC–MS analysis using a GC–MS QP2010 Plus system (Shimadzu, Kyoto, Japan). The system comprised an auto-injector (AOC-20i), a headspace sampler (AOC-20s), a mass selective detector with an ion source set at 220 °C, and a 270 °C interface. The mass range of 40 m/z to 650 m/z was used for analysis, with a threshold of 1000 EV established. Furthermore, the injector was set up for split injection mode with a 10:1 ratio at 260 °C. The temperature was first set to 100 °C for 2 min before being gradually increased to 300 °C at a rate of 10 °C/min. The carrier gas was helium, which had a linear velocity of 40.9 cm/s at a pressure of 90.5 kPa. The system operated at an aggregate flow rate of 16.3 mL/min and a columnar flow rate of 1.21 mL/min. The structure, molecular mass, and spectral fragments were taken into consideration to determine the identity of the molecules, and compounds were matched with the established components from the National Institute of Standards and Technology’s (NIST14s.lib, accessed on 16 March 2023) library database, which contains over 62,000 patterns.

### 2.6. Sample Preparation for Flavonoid Quantification in CdCl_2_ Treated C. tinctorius Tissues by Ultra-High Pressure Liquid Chromatography–Tandem Mass Spectrometry (UHPLC–MS/MS)

Tissues/plant materials of 30–35 mg were weighed. To this, 500 µL of 80% methanol: water (LC–MS grade) was added, and the mixture was ultrasonicated (Aczet Pvt. Ltd., Mumbai, India) at room temperature for 20 min, followed by centrifugation (Eppendorf ^R^ centrifuge 5430 R) at 8000 rpm for 10 min. The supernatant was filtered through a 0.22 µm syringe filter (RanDisc Nylon SF, RANKEM^TM^, Chennai, India) into LC–MS glass vials for vacuum drying in SpeedVac at 65 °C. The dried extract was resuspended in 80% methanol for flavonoid quantification in a UPLC system (Exion LC, Sciex, Gurgaun, India) coupled to a triple quadrupole system (QTRAP6500 + ABSciex, Gurgaun, India) using an electrospray ionization [28]. The voltage was set at 5500 V for positive ionization. For quantitative and qualitative analysis, a mass spectrometer was used in multiple reaction monitoring (MRM) modes using analytical standards of anthocyanidin, flavonols, flavanone, flavanol glycoside, and flavan-3-ols (Merck, Rahway, NJ, USA). The system was used at 70 psi for gas 1 and gas 2, 40 psi for curtain gas, and a 650 °C source temperature in a collision-assisted dissociation medium. Analyst software (version 1.5.2) was used for flavonoid identification and quantification. Details of the flavonoid calibration curve, retention time (min), and MS/MS profile of flavonoids are presented in the Supplementary Files (Figures S1 and S2).

### 2.7. Biochemical Analysis of CdCl_2_-Treated Plants

#### 2.7.1. Protein Estimation

Following Bradford’s method [29], the total protein content for all the treated samples was estimated. About 500 mg of fresh leaf sample was homogenized with 1.5 mL (0.1 M phosphate buffer, pH 7) in a pre-chilled mortar and pestle. The extract was centrifuged at 5000 rpm for 10 min. To 1.0 mL of supernatant, 0.5 mL trichloroacetic acid (TCA) was added and again centrifuged at 5000 rpm for 10 min. After discarding the supernatant, the suspended pellet was washed with chilled acetone and dissolved in 1.0 mL of 0.1 N NaOH. A total of 1.0 mL of Bradford reagent was added to 1.0 mL of aliquot, and its optical density was measured using bovine serum albumin (BSA) as standard at 595 nm.

#### 2.7.2. Proline Estimation

Following the method established by Bates et al. [30], free proline was estimated in all the treated samples. About 200 mg of fresh leaves were homogenized in 4.0 mL of 3% aqueous sulfosalicylic acid and centrifuged at 5000 rpm for 10 min to separate the debris. To 1 mL of the above extract, 1.0 mL of glacial acetic acid and 1 mL of acid ninhydrin were added in sequence, and the reaction mixture was incubated in a bath of boiling water for an hour. The reaction was ceased by placing the mixture in an ice bath. A total of 2 mL of toluene was added to the reaction mixture and stirred well for 20–30 s. The separated red-colored upper toluene layer at room temperature was taken, and its absorbance was measured at 520 nm using L-Proline as standard.

#### 2.7.3. Sugar Estimation

Following the method established by Dey [31], the total sugar content was estimated. A total of 0.5 g of fresh leaves were chopped and placed in a test tube with 10 mL of 70% ethanol. The test tubes were then incubated in an oven for 1 h at 60 °C twice. The final volume increased to 25 mL by adding double-distilled water. A total of 1.0 mL of 5% phenol and 5.0 mL of sulfuric acid (conc.) were added to 1.0 mL of the above aliquot in an ice box and allowed to cool before measuring the optical density. D-glucose (Sigma–Aldrich, St. Louis, MO, USA) was used as a reference standard, and absorbance was measured at 485 nm.

#### 2.7.4. Malondialdehyde (MDA) Estimation

To measure the degree to which lipid peroxidation occurs under heavy metal stress, MDA content was estimated following the method established by Zhou and Leul. A total of 0.5 g in vitro-regenerated leaves of *C. tinctorius* was homogenized in 5.0 mL of 1% TCA and later centrifuged at 8000 rpm for 10 min. To each 0.5 mL of the aliquot of supernatant, TCA (20% with 0.5% TBA) was added and heated at 95 °C for 30 min, followed by immediate cooling in an ice bath. The product was re-centrifuged at 8000 rpm for 15 min, and absorbance was monitored at 450, 532, and 600 nm.

#### 2.7.5. Assessment of Anti-Oxidant Enzyme Activities

A total of 0.1 g leaves of regenerated plantlets were homogenized with the aid of a mortar and pestle using 2.0 mL of 0.1 M extraction buffer (0.5 mM EDTA, 1.0 mM ascorbic acid, and 0.1 M phosphate at 7.5 pH). This plant enzyme extract was centrifuged at 10,000 rpm for 15 min; supernatant was used for further enzyme analysis. The catalase (CAT) activity was estimated by following Aebi’s [32] method by measuring the diminution in absorbance of the reaction mixture (1.0 mL of 0.5 M reaction buffer, Na-phosphate at pH 7.5, 0.1 mL 3.0 mM EDTA, 0.2 mL enzyme extract, and 0.1 mL H_2_O_2_) at 240 nm. The reaction was incubated for 3 min. One unit of CAT activity (U mg^−1^ protein min^−1^) is the enzyme amount required to decompose 1.0 μM of H_2_O_2_ min^−1^. The CAT activity was measured using a coefficient of absorbance of 0.036 mM−1 cm−1. Ascorbate peroxidase (APX) activity was estimated following Nakano and Asada’s [33] method. A reaction mixture of 0.1 ml shoot tissue extract, 1.0 mL 0.1 M sodium buffer at pH 7.2, and 0.1 mL EDTA was made. A total of 1.0 mL of 0.5 mM ascorbate was added to this reaction mixture and allowed to run for 3 min at 25 °C. One unit of APX activity (U mg^−1^ protein min^−1^) corresponds to the enzyme amount required to oxidize 1.0 μmol of ascorbate. The decrease in absorbance at 290 nm due to ascorbate degradation by APX was assessed spectrophotometrically and estimated using a molar extinction coefficient of 2.8 mM^−1^ cm^−1^. Superoxide dismutase (SOD) activity was evaluated by applying Dhindsa’s method [34]. A total of 0.1 g of shoot tissue was homogenized in a 2.0 mL extraction mixture (3 mM EDTA, 1% (*w*/*v*) polyvinylpyrrolidone (PVP), 0.5 M phosphate buffer (pH 7.3), 1.0% (v/v) Triton X100) and centrifuged at 10^4^ rpm at 4 °C for 15 min. The SOD activity of the supernatant was assayed by adding 1.5 mL reaction buffer, 0.2 mL methionine, 3.0 mM EDTA, riboflavin, and 0.1 mL each of 1 M NaCO_3_, 2.25 mM Nitro Blue Tetrazolium (NBT), and enzyme extract. To this, 1.0 mL of double-distilled water was added, and the whole reaction assay was incubated in a test tube for 10 min at 20 °C under two 15 W fluorescent lamps. One enzyme unit expressed as EU mg^−1^ protein is the volume of enzyme extract corresponding to 50% suppression of the photochemical reaction.

### 2.8. Statistical Analysis

Statistical analysis was applied to determine the degree of authenticity of the collected data for various investigated parameters. Each experiment was conducted twice with three replications per set of experiments unless specified otherwise. All the data on in vitro regeneration in the presence of PGRs and varying biochemical and anti-oxidant enzyme activity in response to CdCl_2_ (T0, T1, T2, T3, and T4) are expressed as mean ± standard error. One-way analysis of variance (ANOVA) was carried out. Duncan’s test [35] was used as the post hoc test for the separation of means (*p* < 0.05) using the statistical software IBM SPSS 26.0 (SPSS Inc., Chicago, IL, USA).

## 3. Results

### 3.1. Embryogenic Callus Induction and Shoot Regeneration

**Figure 1:**
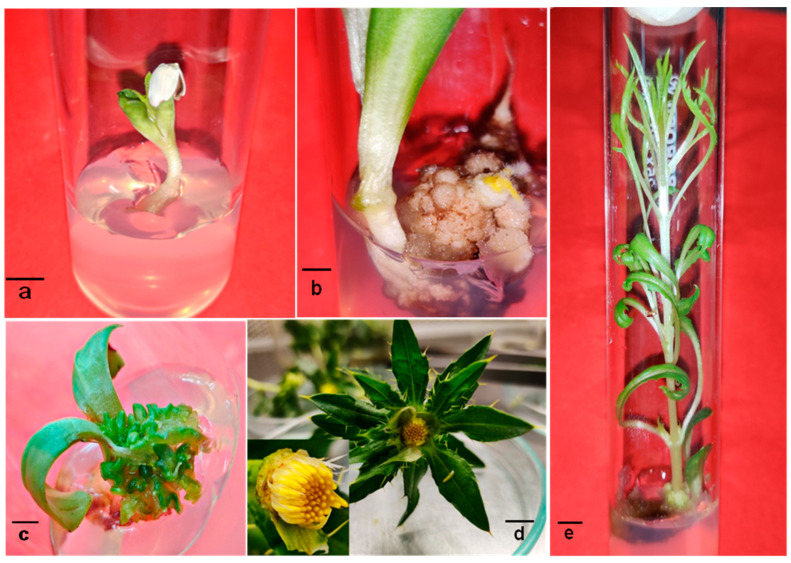
In vitro regeneration of *Carthamus tinctorius* plantlet: in vitro seed germination (**a**); hypocotyl-derived embryogenic callus (**b**); embryo germinated plants (**c**); floral buds on in vitro-regenerated shoot (**d**); elongated in vitro-regenerated shoot (**e**) (Bars (**a**–**c**): 0.5 cm, (**d**,**e**): 1 cm).

Callus was induced from in vitro-germinated seedlings/hypocotyls in a Murashige and Skoog medium (MS) supplemented with 2.2 µM 6-Benzylaminopurine (BAP) + 5.4 µM α-Naphthaleneacetic acid (NAA) + 2.27–4.54 µM thidiazuron (TDZ). The non-embryogenic callus transformed into an embryogenic one after about eight weeks of subculturing. The best treatment for somatic embryo formation was 2.2 µM BAP + 5.4µM NAA + 4.54 µM TDZ, in which 75.6 ± 2.60 embryogenesis frequency (%) with about 20 ± 1.15 somatic embryos per 100 mg callus clump was noted (Table 1). In half-strength MS medium supplemented with 1.40 µM GA3 + 2.2 µM BAP + 5.4 µM NAA, the highest percentage of somatic embryo germination (34 ± 2.31%) was observed. The germinated embryos showed an average of 11 ± 0.57 shootlets with 3 ± 0.57 floral buds in a medium containing 0.54 μM NAA + 9.08 μM TDZ. The plant-regeneration process of *C. tinctorius* is presented in Figure 1a–e.

### 3.2. GC–MS Profiling of Embryogenic Callus and Regenerated Shoot

**Figure 2:**
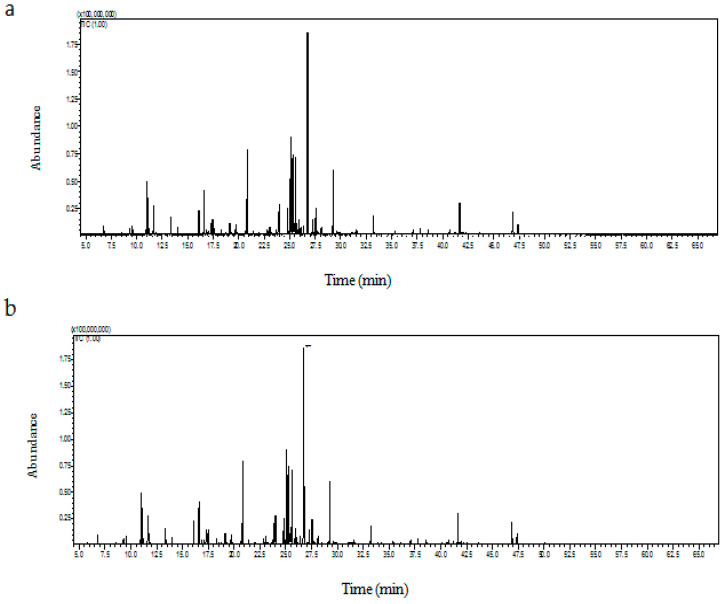
GC–MS profile of total ion chromatogram (TIC) of embryogenic callus (**a**) and in vitro-regenerated shoot (**b**) of *Carthamus tinctorius* L.

Once the embryogenic callus and the regenerated plants developed, we detected the phytocompounds found in these sources. The GC–MS profile of the embryogenic callus (Figure 2a) and the regenerated shoot extract (Figure 2b) indicated the presence of various sugars, sugar acids, sugar alcohols, fatty acids, and organic acids of broad therapeutic value. In callus mass, various organic acids such as α-hydroxyisobutyric acid, 2-furoic acid, 3-hydroxybutyric acid, 3-hydroxyvaleric acid, 4-hydroxybutanoic acid, 3-phenyllactic acid, 4-hydroxybenzoic acid, and 4-hydroxybenzene acetic acid were present exclusively (Table 2). The regenerated shoots had a greater percentage of oxalic acid, methyl succinic acid, malic acid, D-(−)-citramalic acid, 4-aminobutanoic acid, citric acid, and quininic acid compared to the callus mass.

The presence and the amount of carbohydrates (sugar, sugar acid, and sugar alcohol) were different in the callus and the shoot tissue. Sugar alcohols D-fucitol and D-mannitol were present only in the callus, whereas 3-deoxyhexitol and D-glucitol were exclusively present in the regenerated shoot (Table 3). L-threitol, 1-deoxypentitol, and xylitol were more in callus tissue compared to shoots. The fatty acid profiles of both the embryogenic callus and the regenerated shoot revealed the presence of long-chain saturated fatty acids (palmitic acid, C16:0, stearic acid, C18:0), monounsaturated fatty acid (oleic acid, C18:1 and 13-eicosenoic acid, C20:1), polyunsaturated fatty acids (linoleic acid, *C*18:2n6c) and triglyceride (1-monopalmitin). Behenic acid, C22:0, and 1-monomyristin, C14 (monoglyceride), were present only in embryogenic callus, whereas Myristic acid, C14:0, and α-linolenic acid *C*18:3n3 were present only in inflorescence along with above mentioned fatty acids. A maximum percentage of fatty acids were present in inflorescence or capitulum (42.94%), followed by callus (11.75%) and shoot (5.98%). Both callus and shoot followed the same pattern of abundance for fatty acids: 1-monopalmitin > linoleic acid > oleic acid > palmitic acid > stearic acid (Table 4). Among sugars, L-sorbopyranose and ribose were present only in callus while α-D-arabinopyranose, α-D-(+)-talopyaranose, α-D-galactopyranose, β-D-allopyranose and sedoheptulose were present exclusively in the regenerated shoot (Table 5). Among the sugar acids, β-D-glucopyranuronic acid and tartaric acid were present only in the callus, while D-galacturonic acid was present only in regenerated shoots. Gluconic acid was the most abundant sugar acid in callus and shoot tissue (Table 6). The GC–MS of the derivatized sample showed the presence of 17 and 10 amino acids in regenerated shoot and callus tissue, respectively. L-alanine, L-citrulline, L-asparagine, L-ornithine, L-glutamic acid, L-glutamine, and L-tyrosine were found only in the regenerated shoot (Table 7).

**Figure 3:**
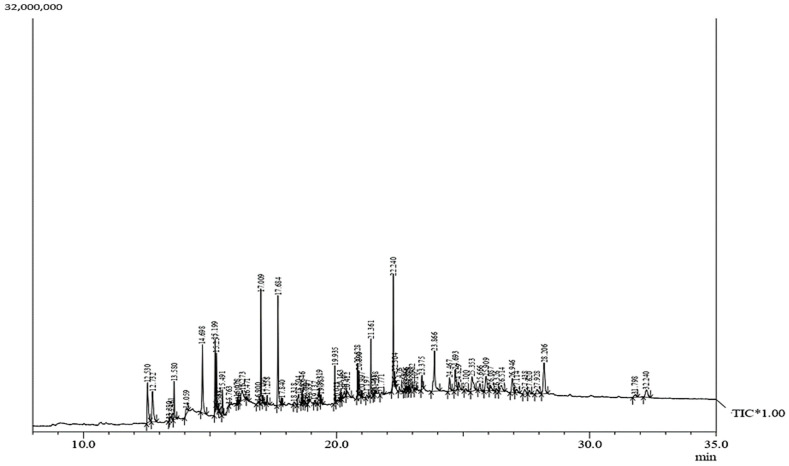
GC–MS-based TIC for metabolomic profiling of phytocompounds present in the regenerated safflower inflorescence; TIC: Total Ion Chromatography, Y-axis: Absorbance and X-axis: Retention time (min).

The phytocompounds, confirmed by the GC–MS spectrum profile of in vitro-regenerated safflower inflorescence (Figure 3), are presented in Table 8. The important unique fatty acids present in the methanolic extract of safflower capitulum were palmitic acid (6.65%), linoleic acid (2.11%), and stearic acid (0.28%). Fatty acid methyl esters (FAME), namely hexadecenoic acid, methyl ester (2.76%), 9,12-octadecadienoic acid (Z,Z) methyl ester (3.51%), 9-octadecenoic acid (Z) methyl ester (2.87%), 11-octadecenoic acid methyl ester (0.18%), N-octadecanoic acid methyl ester (1.40%), and decanoic acid methyl ester (0.27%) were identified.

### 3.3. Quantification of Flavonoids in Embryogenic Callus

As the UHPLC–MS/MS technique provides a full and good view of phytochemicals in plant parts, this technique was used in untreated callus, which showed the presence of flavonoids (<1 ng mg^−1^), naringenin (0.01 ng mg^−1^), myricetin (0.021 ng mg^−1^), kaempferol (0.002 ng mg^−1^), epicatechin gallate (0.048 ng mg^−1^), rutin (0.011 ng mg^−1^), pelargonidin (0.316 ng mg^−1^), peonidin (0.024 ng mg^−1^) and delphinidin (0.078 ng mg^−1^). Though their levels were low (<0.001 ng mg^−1^), the presence of quercetin and cyanidin was also detected (Table 9).

### 3.4. Influence of CdCl_2_ on Flavonoid Accumulation in Regenerated Shoots

**Figure 4:**
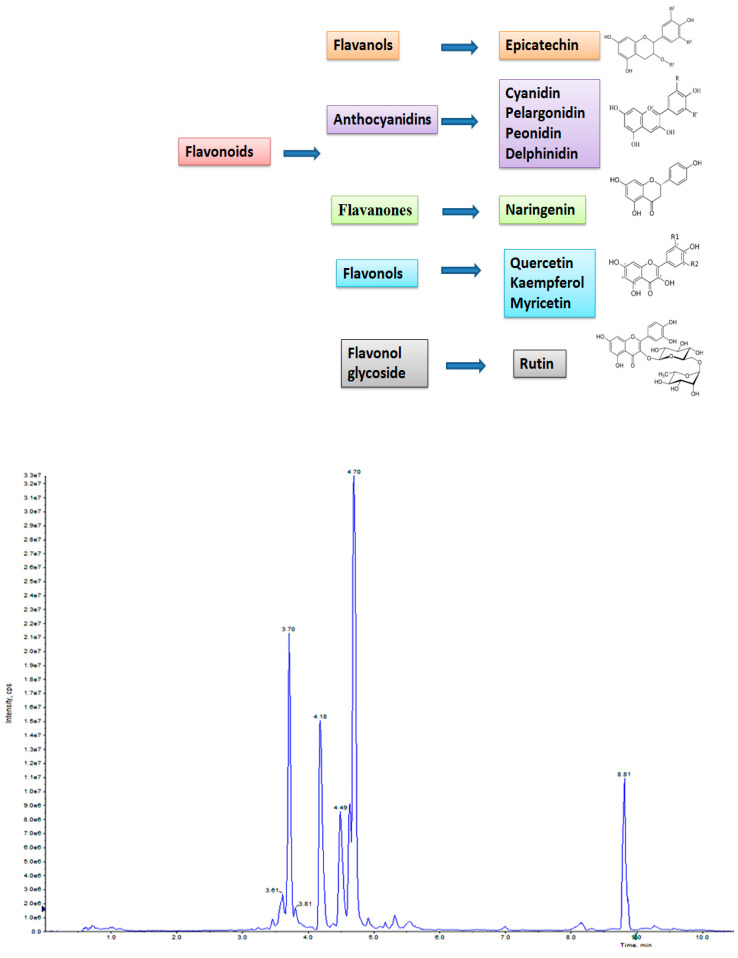
UHPLC–MS/MS total ion chromatogram (TIC) of T0 (control) sample of in vitro-regenerated shoot of safflower showing the presence of various flavonoids.

**Figure 5:**
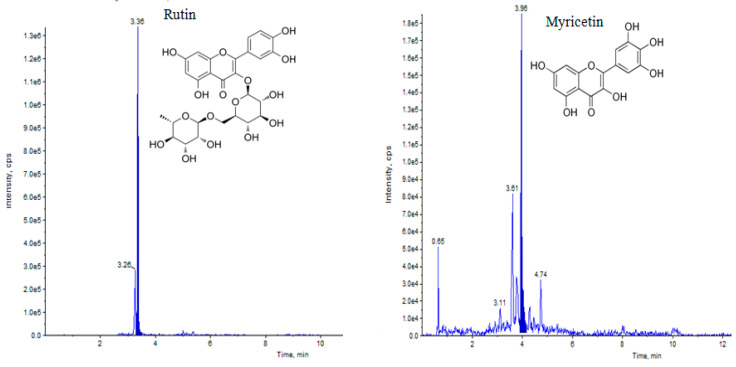
UHPLC–MS/MS (QTRAP 6500) chromatogram of rutin and myricetin flavonoid at retention time 3.36 min and 3.96 min, respectively.

**Figure 6:**
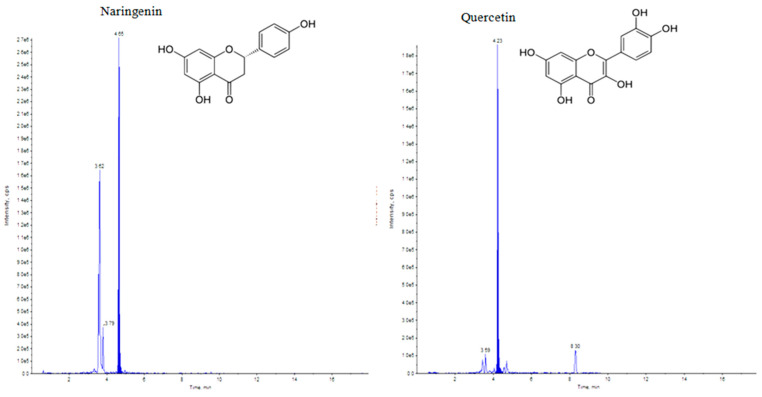
UHPLC–MS/MS (QTRAP 6500) chromatogram of naringenin and quercetin flavonoids at retention time 4.65 min and 4.23 min, respectively.

**Figure 7:**
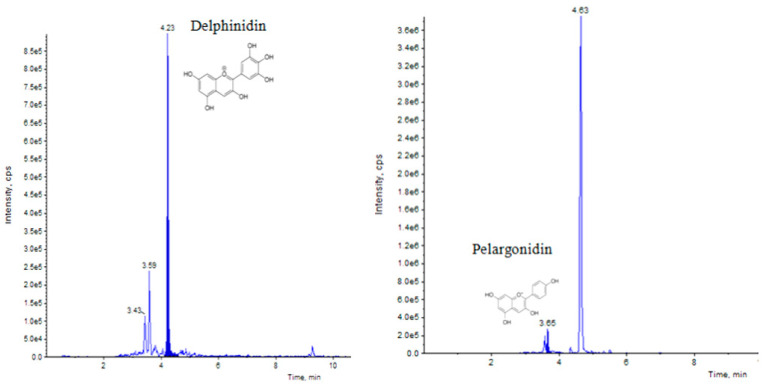
UHPLC–MS/MS (QTRAP 6500) chromatogram of delphinidin and pelargonidin flavonoid at retention time 4.23 min and 3.65 min, respectively.

**Figure 8:**
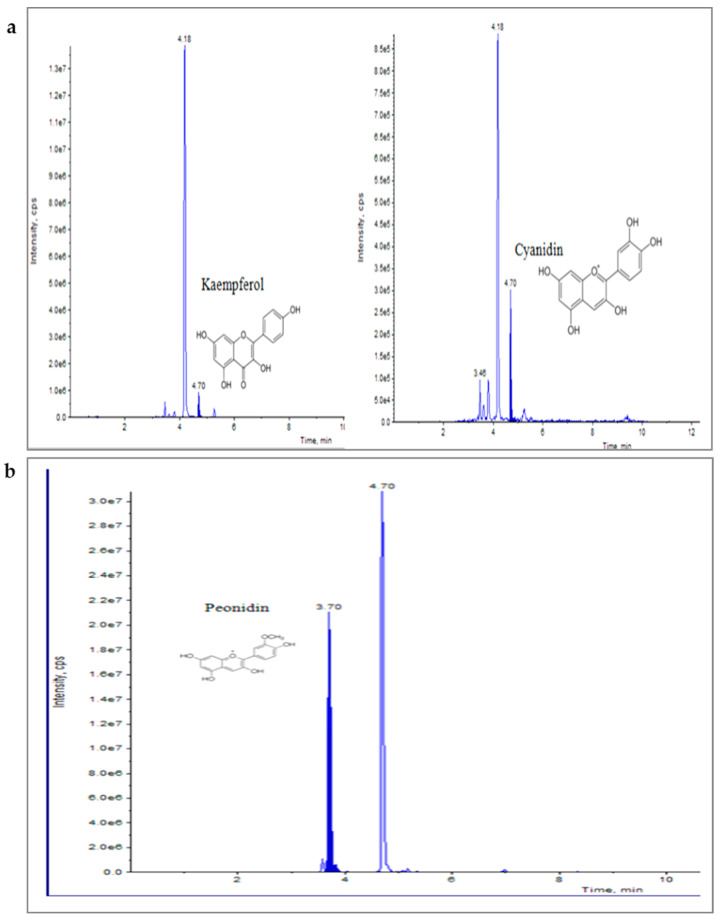
UHPLC–MS/MS (QTRAP 6500) chromatogram of flavonoids, kaempferol and cyanidin (**a**), and Peonidin (**b**) at retention time 4.70 min and 3.70 min, respectively.

Elicitation is an important biotechnological strategy for improving active compounds in plant tissues. These signaling elements of biotic and abiotic nature regulate the cascade of genes in promoting the synthesis of phytocompounds. Here, abiotic elicitor CdCl_2_ was amended in media, and the content of various flavonoids was quantified in in vitro-grown tissues. The flavonoid identification in *C. tinctorius* extracts was made by comparison of retention times, the observation of characteristic MRM transitions, and by matching the MS2 spectra of reference compounds. UHPLC–MS/MS quantification of important flavonoids present in regenerated shoots was made (Figure 4); these were flavan-3-ols (epicatechin gallate), anthocyanidins (cyanidin, pelargonidin, delphinidin, peonidin), flavanones (naringenin), flavonols (quercetin, kaempferol, myricetin), and flavonol glycoside (rutin) (Table 10), of which 6 flavonoids (naringenin, kaempferol, epicatechin gallate, pelargonidin, cyanidin and delphinidin) showed a linear increase with increase in CdCl_2_ dosage, the optimum being at T3 (0.15 mM). A linear yield increase of quercetin (7.07 ng mg^−1^), myricetin (0.46 ng mg^−1^), rutin (1.81 ng mg^−1^) (Figure 5), and peonidin (7.44 ng mg^−1^) was observed with an increase in CdCl_2_ level (0.05 to 0.20 mM CdCl_2_). T4 showed the maximum accumulation of quercetin (Figure 6), myricetin, rutin, and peonidin with about 3.8-, 2.9-, 25.9-, and 8-fold increase in concentration, respectively, as compared to the control mother plant (T0). Flavonoid naringenin (8.68 ng mg^−1^), kaempferol (1.05 ng mg^−1^), epicatechin gallate (0.20 ng mg^−1^), pelargonidin (1.65 ng mg^−1^), cyanidin (1.03 ng mg^−1^) and delphinidin (7.77 ng mg^−1^) (Figure 7 and Figure 8) increased by 6.67-, 1.94-, 3.33-, 2.06-, 1.87-, and 4.415-fold, respectively, at T3. In safflower (T0), the following order of flavonoid abundance was observed: anthocyanidin > flavonols > flavanone > flavanol glycoside > flavan-3-ols.

### 3.5. Amendment of CdCl_2_ and Protein, Proline, Sugar, and Malondialdehyde (MDA) Accumulation

The biochemical attributes of in vitro-regenerated plantlets and calluses were measured post-CdCl_2_ elicitation. After one week of treatment with different dosages of CdCl_2_, the total sugar, protein, and proline content were measured (Table 11). In regenerated plantlets, a linear increase of total protein, proline, and sugar with an increase in elicitor dosage was observed. At T4 (0.20 mM CdCl_2_), the shoots showed maximum content of protein, proline, and sugar (13.26 mg g^−1^ FW; 16.26 µg g^−1^ FW and 17.16 mg g^−1^ FW, respectively). It was noted that the CdCl_2_ stress caused a significant increase in MDA with an increase in CdCl_2_ concentration. Maximum content of MDA (10.26 ± 0.290 nmol g^−1^ FW) was noted at T3 (0.20 mM).

### 3.6. Effect of CdCl_2_ on Anti-Oxidant Enzymatic Activity

**Figure 9:**
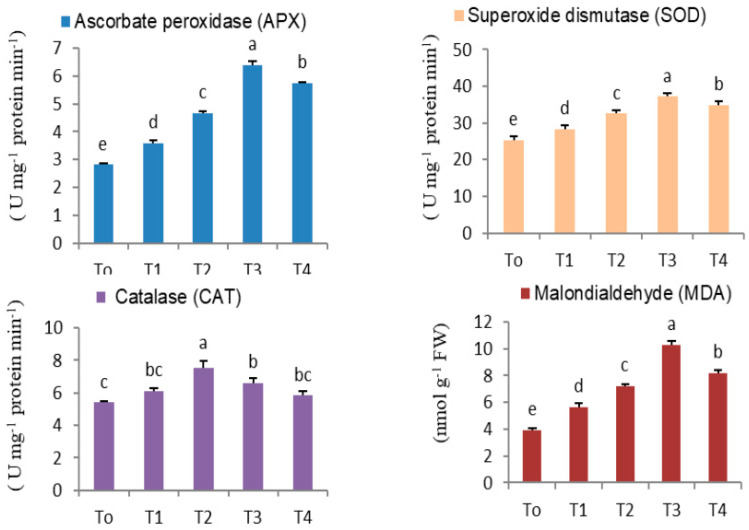
APX, SOD, CAT anti-oxidant enzyme activities and MDA content in in vitro-regenerated safflower shoots after different levels of CdCl_2_ treatment (T0: Control; T1: 0.05; T2: 0.10; T3: 0.15; T4: 0.20 mM). Values are expressed as means ± standard errors of three replicates of two experiments. Means followed by the same letters are significantly different at *p* ≤ 0.05 according to Duncan’s multiple range test (DMRT).

The introduction of abiotic elicitor CdCl_2_ in media induced heavy metal stress in tissue culture, thus triggering reactive oxygen species (ROS) accumulation, boosting the production of secondary metabolites such as phenolics, flavonoids, and anthocyanins, which, along with enzymatic anti-oxidant activity, may enhance the production of ROS. In the present study, the activities of various anti-oxidant enzymes like ascorbate peroxidase (APX), superoxide dismutase (SOD), and catalase (CAT), the enzymatic scavengers of ROS were measured at different dosages of CdCl_2_ (Figure 9). At T3 (0.15 mM), the maximum APX (6.4 ± 0.115 EU min^−1^ mg^−1^ protein) and SOD (37.16 ± 0.881 EU min^−1^ mg^−1^ protein) were observed, whereas a maximum CAT content (7.53 ±0.405 EU min^−1^ mg^−1^ protein) was detected at T2 (0.10 mM).

## 4. Discussion

Plants are sessile organisms growing in the open and are vulnerable to different environmental stressors. To survive the unfavorable environment, plants regulate growth and development by triggering the synthesis and storage of secondary plant metabolites, which have a crucial role in the plant defense system [36]. In the present study, the plants were regenerated through somatic embryos, and the in vitro-grown tissues and organs like shoot, inflorescence, and callus were analyzed through GC–MS for secondary metabolite synthesis. The best somatic embryogenesis was noted in a 2.2 μM BAP + 5.4 μM NAA + 4.54 μM TDZ-added MS medium in which embryogenesis frequency (75.6 ± 2.6%) and somatic embryo numbers (20 ± 1.15) were high. The use of exogenous auxins alone or in conjunction with cytokinin has previously been considered to be an important criterion in developing embryogenic calluses and embryos in culture by accelerating cell division [36]. In a medium supplemented with GA_3_ + BAP + NAA, the embryos germinated, and floral buds developed on plantlets. The methanolic extracts of in vitro-raised plant tissues—embryogenic callus, shoot, and inflorescence—were separately evaluated via GC–MS technology by isolation of phytocompounds present in different tissues, similar to other studies [37]. The GC–MS study revealed that the embryogenic callus had 24 organic acids, 8 sugar alcohols, 7 fatty acids, and 10 amino acids; in regenerated shoots, there were 16 organic acids, 6 sugar alcohols, 5 fatty acids, 5 sugar acids, and 17 amino acids; moreover, 38 different phytocompounds were present in inflorescence. These phytocompounds of different origins show diverse medicinal properties. One such group of protective secondary plant metabolites is a flavonoid, synthesized through the phenylpropanoid pathway [38]. These flavonoids are non-enzymatic ROS scavengers that donate hydrogen atoms directly, inactivating ROS while converting themselves to phenoxy radicals and scavenging other highly reactive molecules [39]. Bioactive metabolites, such as anthocyanins, operate as potent anti-oxidants and metal ion chelators. Thus, exposure of tissues to heavy metals such as Cd acts as stimuli for anthocyanin production [40]. A similar increase in total flavonoid content under various biotic and abiotic stress was reported in several plants [41]. The present results suggest the presence of flavonoids (anthocyanidin, flavonols, flavanone, flavanol glycoside, and flavan-3-ols) with diverse pharmacological properties in *C. tinctorius* under CdCl_2_ stress. The T3 (0.15 mM) level of CdCl_2_ was identified to have a significant stimulatory effect on flavonoid accumulation. Previous studies also suggested that in vitro-applied CdCl_2_ induced the synthesis of pharmacologically significant secondary metabolites like reserpine and ajmalicine in devil pepper (*Rauvolfia serpentina* (L.) Benth. ex Kurz.) [42], alliin in garlic (*Allium sativum* L.) [43], and stevioside and rebaudioside in candyleaf (*Stevia rebaudiana* Bertoni) [44]. UHPLC–MS/MS analysis of embryogenic calluses revealed the presence of pharmacologically important flavonoids; hence, callus culture may be considered to be a promising system for flavonoid production and enrichment. The roles of flavonoids, identified in in vitro-regenerated safflower shoots and embryogenic callus, are summarized in Table 12.

The phytocompounds identified in the spectrum profile of the methanol extract of callus, shoots, and inflorescence of *C. tinctorius* confirmed that these compounds are promising candidates for therapeutic use. Among the important bioactive compounds detected, vitamin E can cure leukemia, tumors, cancer, dermatitis, ulcers, and inflammation. It has anti-aging, analgesic, antidiabetic, vasodilator, antispasmodic, antibronchitic, antiplasmodial, antimicrobial, and anti-inflammatory properties [55]. The 9, 12-octadecadienoic acid is beneficial in the treatment of inflammation, microbial illness, and arthritis [56]. Linoleic acid and oleic acid have antibacterial properties. Long-chain fatty alcohol n-nonadecanol-1 also showed antimicrobial properties. The straight-chain primary alcohol 1-heptacosanol acts as a flavoring and fragrance compound, which may reduce cholesterol levels, and it has antimicrobial, cytotoxic, and antithrombotic properties. Free fatty acids like long-chain unsaturated fatty acids have been reported to have antimicrobial, anti-inflammatory, and antifungal properties. The anticarcinogenic and anti-oxidant properties of 9-octadecenoic acid (Z)-, methyl ester, and heptacosane have also been described. Various hexadecenoic acid methyl ester activities like hypocholesterolemic, antifungal, anti-oxidant, antibacterial, nematicide, pesticide, anti-androgenic, flavor, hemolytic, and 5-alpha reductase’s inhibitory properties have previously been reported. Fatty acid methyl esters (FAME) with methanol identified in the GC–MS chromatogram of safflower extract are used in the production of biodiesel and detergents. The different biomolecules in safflower callus and in vitro-regenerated shoots and inflorescence with significant biological capabilities were established in this investigation, validating the medicinal and therapeutic uses of *C. tinctorius*.

Plants possess a natural defense system, including osmoprotectants (sugar, protein, proline, etc.) and enzymatic anti-oxidants (APX, SOD, and CAT) for protection against oxidative damage triggered by multitudinous environmental stresses. Plants can avoid dehydration by lowering osmotic potential, including enhancing the synthesis, accumulation, compartmentalization, and transport of organic osmolytes. Osmolytes, like soluble sugar, protein, and proline, play important roles in osmotic adjustments under heavy metal stress. The present research investigated the impact of CdCl_2_ stress on biochemical attributes in safflower. In response to induced abiotic stress, increased cytosolic proline, protein, sugar, and MDA content was observed at a high elicitor dose (T4). Increased proline level was seen as a physiological adaptation of plants to heavy metal stress to protect the cellular architecture [57]. Proline acts as a metal-chelating osmolyte, resulting in decreased phytotoxicity, and it is an anti-oxidant defense and messenger molecule. In addition, it may play a role in improving the stability and integrity of the biological membrane protein. Accumulated proline, acid amine, and osmotic substances neutralize the effect of heavy metal stress in plants. They also aid enzyme activity by increasing plant water uptake potential and can suppress programmed cell death. Elevated levels of proline in response to Cd stress have also been documented in many plants like chickpea (*Cicer arietinum* L.), devil pepper (*Rauvolfia serpentina*), garlic (*Allium sativum*), maize (*Zea mays* L.), and summer savory (*Satureja Hortensis* L.) [50,58]. Proteins make plants competent against heavy metal stress through various mechanisms involving ROS degeneration, metal sequestration in storage organelles, regulation of metallo-enzymes, and activation of genes involved in transcription [59]. Metallothioneins (MTs) are low-molecular-weight cysteine-rich proteins whose thiol group binds metals via mercaptide bonds and regulates metal metabolism in alleviating metal toxicity. Heavy metals such as Cd, Cu, Zn, Hg, Ni, and Co can trigger the transcriptional regulation of MT biosynthesis. Since sugar functions as an osmoprotectant and shields cellular membranes, high sugar levels were detected at elevated CdCl_2_ doses. This variation in soluble sugar concentration makes plants more tolerant to abiotic stress by altering signaling pathways, triggering the production of repair enzymes and effective ROS scavengers [60]. Following exposure to heavy metal stress, malondialdehyde (a cytotoxic byproduct of lipid peroxidation; MDA) increased because of membrane destabilization and enhanced free radical production, as reported in many plants [61,62,63,64]. In this safflower study, MDA content increased linearly till T3, and then it declined, as was observed in other plants under Cd stress.

Reactive oxygen species target biomolecules (proteins, lipids, and DNA) at various locations, triggering oxidation and altering their functional and structural properties [65,66]. Modifications of proteins may increase in plants subject to a variety of stress factors. Plants have evolved a complex anti-oxidant defense system that includes low-molecular-mass anti-oxidants like ascorbate, reduced glutathione, tocopherol, carotenoids, and flavonoids, along with anti-oxidant enzymes like superoxide dismutase (SOD), ascorbate peroxidase (APX), and catalase (CAT) to mitigate the adverse effects of oxidative stress. Several studies indicated that anti-oxidants have a defensive role against Cd stress in plants [67]. Cadmium indirectly boosts ROS production by interfering with electron transport, a pivotal event in photosystem II. In the present study, SOD activity in CdCl_2_-treated tissues increased linearly up to 0.15 mM (T3 treatment), beyond which (0.20 mM) a marginal decline was noted. A decline in SOD at high dose (T4) may be attributed to H_2_O_2_-mediated SOD inactivation, produced through enzymatic and non-enzymatic pathways in various cell compartments [68]. A similar response was noted in safflower and sunflower calluses in response to Cd (10–100 µM). Since SOD converts superoxide radical (O^2−^) to H_2_O_2_ and O_2_, its presence is proven to boost the defense mechanism in CdCl_2_-stressed cells, and thus SOD is more effective in quenching ROS. A similar increase in SOD activity was recorded in soybean (*Glycine max* (L.) Merr.), garlic *(Allium sativum*), in fenugreek (*Trigonella foenumgraecum* L.). APX and CAT are representative heme enzymes counteracting oxidative stress; CAT, in particular, is the main enzyme responsible for the catalytic scavenging of H_2_O_2_ into oxygen along with water in plant peroxisomes. Under CdCl_2_ stress, CAT activity elevated concurrently until T2, while APX activity increased until T3. Increased CAT and APX activity under abiotic stresses has been reported in several plants that have been studied, such as cucumber (*Cucumis sativus* L.) and garlic (*Allium sativum*) [69,70]. The decrease in CAT after T2 treatment could be attributed to the inhibitory action of nitric oxide (NO), which accumulates in peroxisomes in the presence of heavy metals and other abiotic stressors, as previously reported in arabidopsis (*Arabidopsis thaliana* (L.) Heynh.). On exposure to the elicitor (CdCl_2_), anti-oxidant enzyme activity increased, indicating induced oxidative stress. This suggests that CdCl_2_ elicitation is a promising strategy for inducing stress and may be utilized to produce secondary metabolites in plant cultures.

## 5. Conclusions

It is evident from the present GC–MS study that a diverse range of phytocompounds is present in embryogenic callus, regenerated shoots, and the inflorescence of in vitro-regenerated plants. This safflower plant may be used for large-scale production of pharmacologically active flavan-3-ols (epicatechin gallate), anthocyanidins (cyanidin, pelargonidin, delphinidin, peonidin), flavanones (naringenin), flavonols (quercetin, kaempferol, myricetin) and flavonol glycoside (rutin). The UHPLC–MS/MS analysis revealed that the addition of low CdCl_2_ doses (0.15–0.20 mM) efficiently enhanced several compounds, including a wide array of flavonoids. The amendment of CdCl_2_ induced cellular stress in culture. Biochemical attributes like protein, proline, sugar, malondialdehyde, and anti-oxidant enzyme activities (APX, CAT, and SOD) were elevated in ameliorating stress. The amendment of CdCl_2_ thus could be a practical approach for enhancing secondary metabolite synthesis in safflower. A thorough investigation of GC–MS and UHPLC–MS/MS identified phytochemicals, and an assessment of biological importance and toxicity will yield novel drugs with beneficial activity.

## Figures and Tables

**Table 1:** Somatic embryo formation, germination, and plantlet regeneration in different plant growth regulators (PGRs) added to full-strength MS medium (F-MS).

F-MS Medium + PGRs (µM)				Embryogenesis % (after 8-Week of Subculturing)	Mean No. of Somatic Embryos per Callus Clump (100 mg)
BAP	TDZ	NAA			
2.2	5.4	2.27		40 ± 1.15 ^c^	10.33 ± 0.88 ^b^
2.2	8.0	2.27		49.33 ± 1.76 ^b^	13.33 ± 1.76 ^b^
2.2	5.4	4.54		75.6 ± 2.60 ^a^	20 ± 1.15 ^a^
				**Germination frequency (%)** **of somatic embryos**	
2.2	5.4	-	½ MS + 0.29	27.60 ± 0.88 ^b^	
2.2	5.4	-	½ MS + 1.40	34.00 ± 2.31 ^a^	
2.2	5.4	-	1.40	21.33 ± 1.76 ^b^	
				**No. of somatic** **embryo regenerated shoots**	**Mean no. of** **flower buds per plantlet**
-	0.54	4.54		5.33 ± 0.88 ^b^	0 ^c^
-	0.54	9.08		11 ± 0.57 ^a^	3 ± 0.57 ^a^
2.2	0.54	4.54		6.33 ± 0.33 ^b^	1.33 ± 0.33 ^b^

Values are Mean ± SE of three replicas of two experiments. Means in the column followed by the same superscript are not significantly different according to DMRT at *p* ≤ 0.05.

**Table 2:** Organic acid trimethylsilyl (TMS) derivatives present in embryogenic callus and in vitro-regenerated shoot of *Carthamus tinctorius* as identified by GC–MS.

S.No.	Retention Time (Rt)	*m*/*z*	Organic Acids	Area% Callus	Area % Plantlet
1	5.78	73	Lactic Acid, 2TMS	0.836	0.391
2	5.865	73	α-Hydroxyisobutyric acid, 2TMS	0.062	-
3	6.148	147	Glycolic acid, 2TMS	0.543	0.248
4	7.563	125	2-Furoic acid, 1TMS	0.0203	-
5	7.672	73	Oxalic acid, 2TMS	0.165	0.182
6	7.774	147	Hydracrylic acid, 2TMS	0.259	0.163
7	8.170	147	3-Hydroxybutyric acid, 2TMS	0.079	-
8	9.279	147	Propanedioic acid, 2TMS	1.973	1.586
9	9.431	147	3-Hydroxyisovaleric acid, 2TMS	0.079	-
10	10.124	147	4-Hydroxybutanoic acid, 2TMS	0.055	-
11	12.154	147	Butanedioic acid, 2TMS	0.827	0.410
12	12.452	147	Methyl succinic acid, 2TMS	0.020	0.028
13	12.562	73	Glyceric acid, 3TMS	0.451	0.371
14	12.866	147	Itaconic acid, 2TMS	0.392	0.160
15	13.595	84	Malic acid, 3TMS	10.18	13.54
16	16.095	73	D-(−)-Citramalic acid, 3TMS	0.136	6.881
17	16.768	84	Pyroglutamic acid, TMS	0.200	0.102
18	17.523	174	4-Aminobutanoic acid, 3TMS	0.293	4.850
19	18.742	73	3-Phenyllactic acid, 2TMS	0.313	-
20	19.815	73	4-Hydroxybenzoic acid, 2TMS	1.215	-
21	20.062	73	4-Hydroxybenzene acetic acid, 2TMS	0.051	-
22	23.550	75	Azelaic acid, 2TMS	0.413	0.099
23	23.995	73	Citric acid, 4TMS	0.798	9.294
24	24.792	73	Quininic acid, 5TMS	1.009	8.198

**Table 3:** Sugar alcohol trimethylsilyl (TMS) derivatives present in embryogenic callus and in vitro-regenerated shoot of *Carthamus tinctorius* as identified by GC–MS.

S. No.	Retention Time	*m*/*z*	Sugar Alcohol	Area % Callus	Area % Plant
1	16.866	73	L-Threitol, 4 TMS	0.534	0.2679
2	17.749	73	1-Deoxypentitol, 4 TMS	2.710	0.100
3	21.387	73	Xylitol, 5TMS	3.04	1.596
4	23.281	73	D-Fucitol, 5 TMS	0.789	-
5	23.424	73	3-Deoxyhexitol, 5TMS	-	0.123
6	25.439	73	D-Glucitol, 6TMS	-	4.551
7	29.235	73	Myo-inositol, 6TMS	2.307	8.637
8	40.174	73	D-Mannitol, 6TMS	0.374	-
9	40.353	73	D-Sorbitol, 6TMS	0.264	-
10	41.150	73	D-Galactitol, 6TMS	0.348	-

**Table 4:** Fatty acid trimethylsilyl (TMS) derivatives present in embryogenic callus and in vitro-regenerated shoot of *Carthamus tinctorius* as identified by GC–MS.

S.No.	Retention Time	*m*/*z*	Fatty Acid	Area % Callus	Area % Plant
1	28.494	73	Palmitic acid, 1TMS	1.179	0.600
2	31.442	73	Oleic acid, 1TMS	1.623	1.144
3	31.562	73	Linoleic acid, 1TMS	3.383	1.959
4	32.039	117	Stearic acid, 1TMS	0.421	0.188
5	34.561	343	1-Monomyristin, 2TMS	0.152	-
6	37.771	371	1-Monopalmitin, 2TMS	4.7069	2.087
7	38.722	117	Behenic acid, 1TMS	0.2879	-

**Table 5:** Sugar trimethylsilyl (TMS) derivatives present in embryogenic callus and in vitro-regenerated shoot of *Carthamus tinctorius* as identified by GC–MS.

S.No.	Retention Time	*m*/*z*	Sugar	Area % Callus	Area % Plant
1	20.249	73	α-D-Arabinopyranose, 4TMS	-	0.171
2	23.899	73	L-Sorbopyranose, 5TMS	1.160	-
4	25.694	204	α-D-(+)-Talopyranose, 5TMS	-	0.340
5	28.194	73	Glucopyranose, 5TMS	1.77	9.465
6	28.817	204	α-D-Glactopyranose, 5TMS	-	0.362
7	29.360	73	N-Acetyl-D-galactosamine, 4TMS	1.708	0.168
8	32.156	204	D-Galactose, 5TMS	0.326	0.229
9	34.392	73	Ribose, 4 TMS	0.126	-
10	36.474	73	D-Xylopyranose, 4TMS	0.093	0.210
11	39.852	204	Lactose, 8TMS	0.269	2.171
12	41.040	73	D-Trehalose, 7 TMS	1.242	0.693
13	42.479	73	β-D-Allopyranose, 5TMS	-	0.2725
14	45.028	73	Sedoheptulose, 6TMS	-	0.277

**Table 6:** Sugar acid trimethylsilyl (TMS) derivatives present in embryogenic callus and in vitro-regenerated shoot of *Carthamus tinctorius* as identified by GC–MS.

S.No.	Retention Time	*m*/*z*	Sugar Acid	Area % Callus	Area % Plant
1	12.562	73	Glyceric acid, 3TMS	0.451	0.371
2	19.096	73	Tartaric acid, 4TMS	0.429	-
3	27.716	73	Galactaric acid, 6TMS	0.893	1.447
4	27.554	73	D-Gluconic acid, 6TMS	5.916	10.92
5	37.252	73	D-Galacturonic acid, 5TMS	-	0.4294
6	45.803	73	β-D-Glucopyranuronic acid, 5TMS	0.4838	-

**Table 7:** Amino acid trimethylsilyl (TMS) derivatives present in embryogenic callus and in vitro-regenerated shoot of *Carthamus tinctorius* as identified by GC–MS.

S.No.	Retention Time	*m*/*z*	Amino Acid	Area % Callus	Area % Plant
1	6.46	72	L-Valine, TMS	0.110	0.098
2	6.767	248	L-Alanine, 3TMS	-	2.405
3	8.545	70	L-Proline, TMS	0.053	0.758
4	8.609	86	L-Isoleucine, TMS	0.104	0.052
5	10.642	73	L-Serine, 2TMS	0.252	0.313
6	13.591	73	L-Citrulline, 4TMS	-	0.183
7	13.993	73	L-Threonine, 3TMS	0.227	2.037
8	14.934	73	L-Aspartic acid, 2TMS	0.206	0.210
9	15.026	248	Beta-Alanine, 3TMS	0.080	0.221
10	16.768	84	Pyroglutamic acid, TMS	0.200	0.102
11	19.125	44	L-Asparagine, 2TMS	-	4.265
12	19.568	73	L-Ornithine, 3TMS	-	0.339
13	19.685	246	L-Glutamic acid, 3TMS	-	1.818
14	19.725	73	Phenylalanine, 2TMS	0.789	2.845
15	23.038	156	L-Glutamine, 3TMS	-	2.708
16	26.08	73	L-Lysine, 4TMS	0.499	2.246
17	26.383	218	L-Tyrosine, 3TMS	-	3.363

**Table 8:** GC–MS metabolomic profiling of phytocompounds present in in vitro-regenerated inflorescence of *Carthamus tinctorius.*

S.No.	Retention Time	Area %	Phytocompound
1	12.530	3.72	Deca-4,6-diyn-1-yl 3-methylbutanoate
2	12.732	4.07	(E)-Deca-8-en-4,6-diyn-1-yl 3-methylbutanoate
3	13.580	2.76	Hexadecanoic acid, methyl ester
4	14.698	6.65	Palmitic Acid, TMS derivative
5	15.199	3.51	9,12-Octadecadienoic acid (Z,Z)-, methyl ester
6	15.257	2.87	9-Octadecenoic acid (Z)-, methyl ester
7	15.312	0.18	11-Octadecenoic acid, methyl ester
8	15.491	1.40	N-Octadecanoic acid methyl ester
9	16.273	2.11	9,12-Octadecadienoic acid (Z,Z)-, TMS derivative
10	16.471	0.28	Stearic acid, TMS derivative
11	17.009	5.98	Heneicosane
12	17.684	6.99	Nonacosane-6,8-dione
13	18.897	0.27	Docosanoic acid, methyl ester
14	19.935	1.84	Eicosyl trifluoroacetate
15	20.828	1.69	Pentacosane-6,8-dione
16	20.890	1.52	Hexacosane-4,6-dione
17	20.997	0.31	Squalene
18	21.361	3.06	1-eicosanol
19	22.240	6.60	Heptacosane-6,8-dione
20	22.304	0.54	Tricosane-4,6-dione
21	22.476	0.39	Ergost-4,7,22-trien-3.alpha.-ol
22	22.756	0.33	1-Heptacosanol
23	22.818	0.21	1-Docosanol
24	23.102	0.17	1-Eicosanol
25	23.375	1.17	Vitamin E
26	23.866	5.09	Nonacosane-6,8-dione
27	24.467	1.47	Isotomatidine, N-acetyl-
28	24.693	2.37	Stigmasta-5,22-dien-3-ol
29	25.353	2.65	gamma.-Sitosterol
30	25.909	2.14	beta.-Amyrin
31	26.514	1.21	Methyl commate d
32	26.946	1.91	Olean-12-en-3-ol, acetate, (3.beta.)-
33	27.438	0.85	Phytyl palmitate
34	27.620	0.44	24-Norursa-3,12-diene
35	27.928	0.81	Lupeol
32	26.946	1.91	Olean-12-en-3-ol, acetate, (3.beta.)-
33	27.438	0.85	Phytyl palmitate
35	27.928	0.81	Lupeol
36	28.206	4.49	Phytyl tetradecanoate
37	31.798	0.47	Isopropyl lineolate
38	32.240	1.49	Bicyclo[10.8.0]eicosan, trans-

**Table 9:** UHPLC–MS/MS quantification of flavonoids present in eight-week-old embryogenic callus of *Carthamus tinctorius.*

S. No.	Flavonoid Subclass	Flavonoids	*m*/*z* (Da) for Q1/Q3	Retention Time	Concentration of Analyte (ng/mg) in Callus Extract
1	Flavanone	Naringenin	273/153	4.65	0.01
2	Flavonols	Quercetin	303/153	4.28	ND
3	Flavonols	Myricetin	319/153	3.97	0.021
4	Flavonols	Kaempferol	287/153	4.74	0.002
5	Flavan-3-ols	Epicatechin gallate	443/139	2.99	0.048
6	Flavonol glycoside	Rutin	611/303	3.36	0.011
7	Anthocyanidin	Pelargonidin	271/197	3.65	0.316
8	Anthocyanidin	Peonidin	301/286	3.49	0.024
9	Anthocyanidin	Cyanidin	287/213	4.74	ND
10	Anthocyanidin	Delphinidin	303/257	4.23	0.078

**Table 10:** UHPLC–MS/MS quantification of flavonoids in safflower leaves under various levels of CdCl_2_ treatment after one week of exposure.

S.No	Flavonoids	Rt	Analyte Concentration (ng/mg)					Fold Increase in Analyte Concentration
			T0	T1	T2	T3	T4	Tmax/T0
1	Naringenin	4.65	1.30	6.40	6.74	8.68	7.53	6.67
2	Quercetin	4.28	1.86	2.38	2.54	3.67	7.07	3.80
3	Myricetin	3.97	0.16	0.33	0.35	0.45	0.46	2.875
4	Kaempferol	4.18	0.54	0.60	0.86	1.05	0.83	1.94
5	Epicatechin gallate	2.99	0.06	0.07	0.19	0.20	0.15	3.33
6	Rutin	3.36	0.07	1.05	1.36	1.63	1.81	25.857
7	Pelargonidin	3.65	0.80	0.96	1.07	1.65	1.42	2.06
8	Peonidin	3.49	0.93	1.06	1.08	1.12	7.44	8.00
9	Cyanidin	4.74	0.55	0.57	1.01	1.03	0.76	1.87
10	Delphinidin	4.23	1.76	2.43	2.60	7.77	3.52	4.415

**Table 11:** Protein, proline, and sugar levels in leaves of in vitro-regenerated shoot in different CdCl_2_ added medium.

CdCl_2_ Treatment	Protein (mg g^−1^ FW)	Proline (µg g^−1^ FW)	Sugar (mg g^−1^ FW)
Callus	2.21 ± 0.036 ^f^	1.07 ± 0.04 ^f^	5.32 ± 0.144 ^f^
T_0_	5.56 ± 0.072 ^e^	5.326 ± 0.263 ^e^	7.106 ± 0.206 ^e^
T_1_	7.063 ± 0.131 ^d^	8.353 ± 0.389 ^d^	10.373 ± 0.287 ^d^
T_2_	9.53 ± 0.44 ^c^	11.613 ± 0.357 ^c^	13.166 ± 0.22 ^c^
T_3_	11.51 ± 0.158 ^b^	14.28 ± 0.601 ^b^	15.716 ± 0.116 ^b^
T_4_	* 13.26 ± 0.29 ^a^	* 16.26 ± 0.29 ^a^	* 17.16 ± 0.375 ^a^

* Represents highest value. Values are means ± standard errors of 3 replicates of two experiments. Within each row, means followed by the same letter are significantly different at *p* ≤ 0.05, according to DMRT. CdCl_2_ elicitor treatment dose: Control (T0), 0.05 mM (T1), 0.10 mM (T2), 0.15 mM (T3), 0.20 mM (T4); Treatment period—1 week.

**Table 12:** Pharmacological roles of flavonoids identified in in vitro-regenerated safflower leaves and embryogenic callus.

S.No.	Flavonoid	Pharmacological Uses	References
1	Naringenin	Anti-atherosclerosis, cardioprotective, anti-inflammatory, anti-oxidant, anti-hyperglycemic, anticancer, antifibrosis	[45]
2	Quercetin	Antidiabetic, anticancer, antifungal, anti-oxidant, anti-obesity, anti-viral, anti-inflammatory, antibacterial	[46]
3	Myricetin	Immunomodulatory activity, cardio-cerebrovascular protection, anti-neurodegenerative, antidiabetic, antimicrobial, Inhibition of pulmonary fibrosis and gastric acid secretion, hepatoprotective, strengthens bones, anti-obesity	[47]
4	Kaempferol	Protection of heart function, vascular endothelium, cranial nerve, against liver injury and metabolic disorders, anti-inflammation, anticancer, treatment for fibroproliferative disorders	[48]
5	Epicatechin gallate	Anti-inflammation, anti-oxidant, growth inhibition, suppress metastasis	[49]
6	Rutin	Anti-inflammatory, anticancer, antidiabetic, anti-allergic, cardioprotective	[50]
7	Pelargonidin	Anti-oxidant, anticancer, anti-inflammatory, detoxification, cardiovascular protection,	[51]
8	Peonidin	Chemopreventive, anti-inflammatory, anti-oxidant	[50]
9	Cyanidin	Anti-oxidant, radical-scavenging activity, anticancer, anti-hyperglycemic, anti-inflammatory	[52]
10	Delphinidin	Anti-inflammatory, anti-oxidation, antimicrobial, antidiabetic, cardiovascular protection, neuroprotection, anticancer, anti-adipogenesis	[53,54]

## Data Availability

The data used to support the findings of this study are not publicly available due to privacy or ethical restrictions, may be obtained from the corresponding author upon request.

## Supplementary Materials

The following supporting information can be downloaded at: https://www.mdpi.com/article/10.3390/metabo14020127/s1, Figure S1: Flavonoid calibration curve for quantification of analyte by UHPLC-MS/MS; Figure S2: MS/MS chromatograms of target flavonoids.
