# Supplementary material for: Phytocompounds and Regulation of Flavonoids in In Vitro-Grown Safflower Plant Tissue by Abiotic Elicitor CdCl2
Source: Metabolites. 2024 Feb 16;14(2):127. doi: 10.3390/metabo14020127 (PMC10891796; doi:10.3390/metabo14020127)

**Figure S1.** Flavonoid concentrations and calibration curve for quantification of analytes by UHPLC-MS/MS. From top to bottom: Quercetin, Rutin, Myricetin, Epicatechingallate, Kaempferol, Cyanidin, Delphinidin, Pelargonidin, Narenginin and Paonidin.

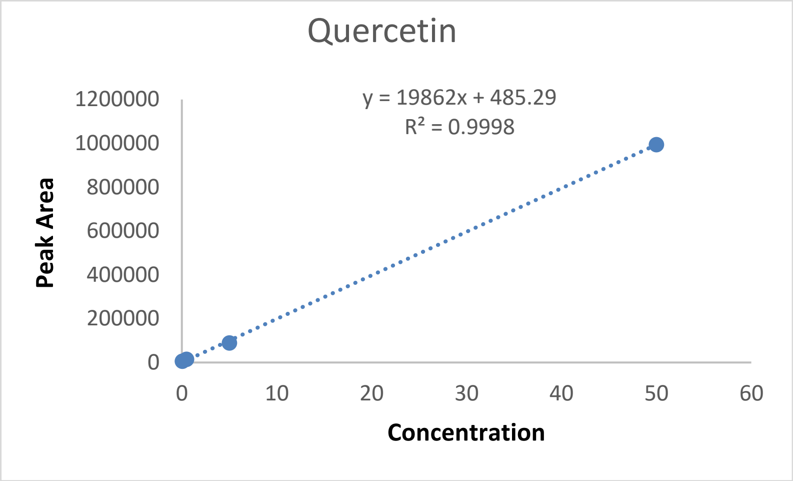


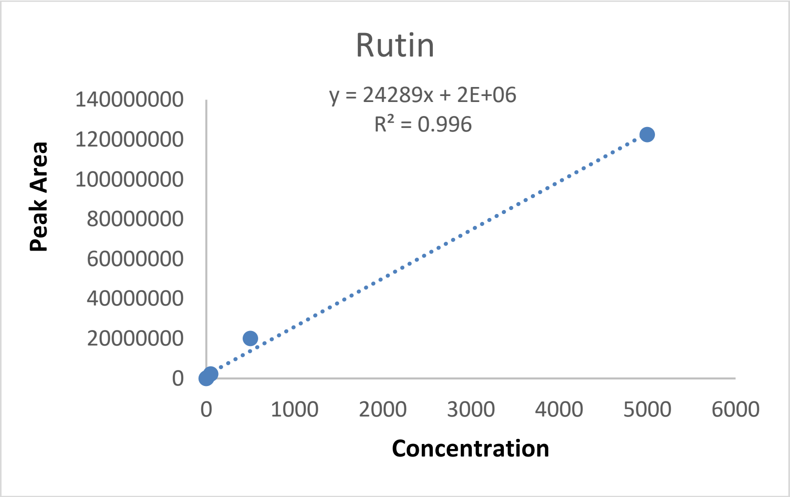


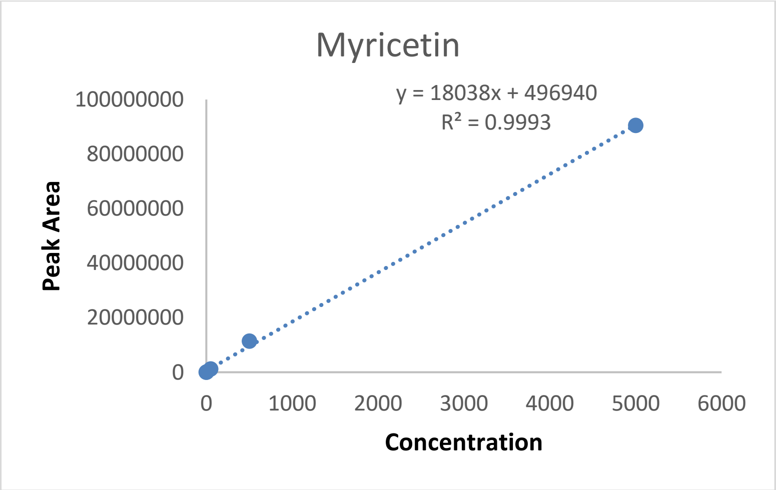

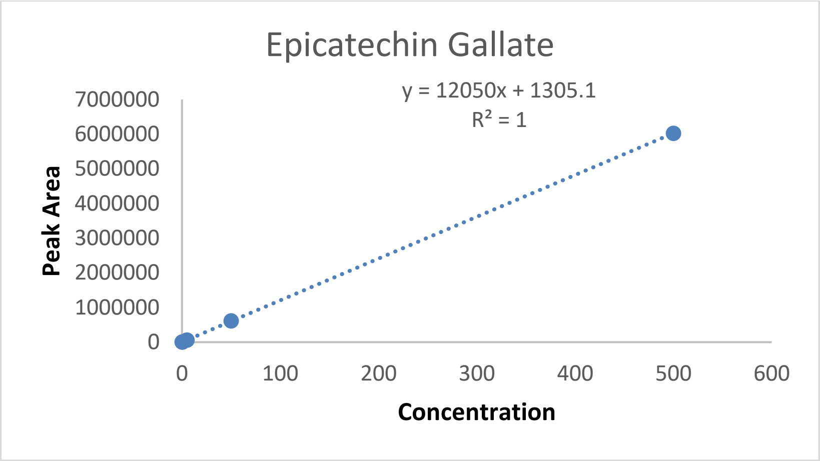
Epicatechin Gallate
y = 12050x + 1305.1
R² = 1
Peak Area
7000000
6000000
5000000
4000000
3000000
2000000
1000000
0
0
100
200
300
400
500
600
Concentration

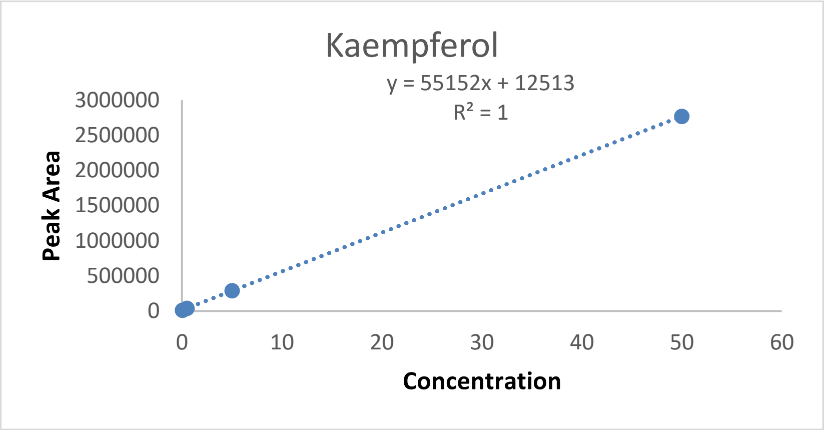
Kaempferol
y = 55152x + 12513
R² = 1
Peak Area
3000000
2500000
2000000
1500000
1000000
500000
0
0
10
20
30
40
50
60
Concentration

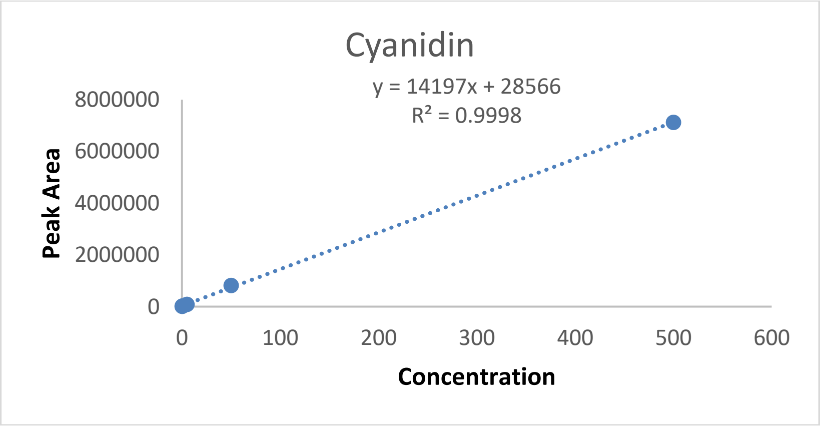
Cyanidin
y = 14197x + 28566
R² = 0.9998
Peak Area
8000000
6000000
4000000
2000000
0
0
100
200
300
400
500
600
Concentration

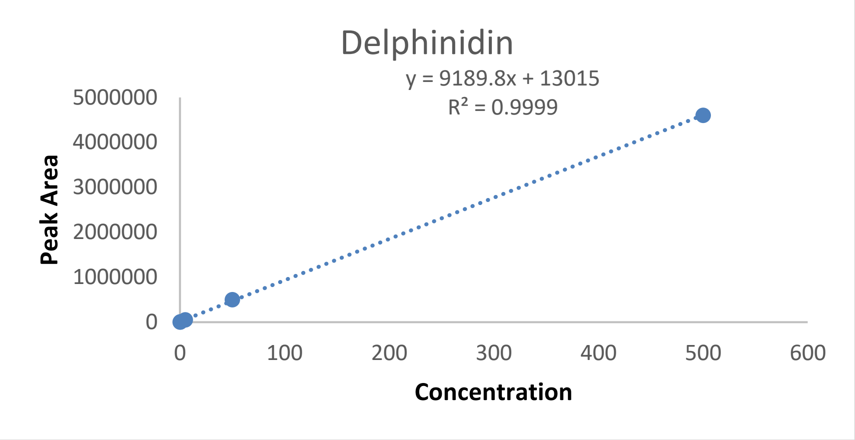
Delphinidin
y = 9189.8x + 13015
R² = 0.9999
5000000
4000000
3000000
2000000
1000000
0
0
100
200
300
400
500
600
Peak Area
Concentration

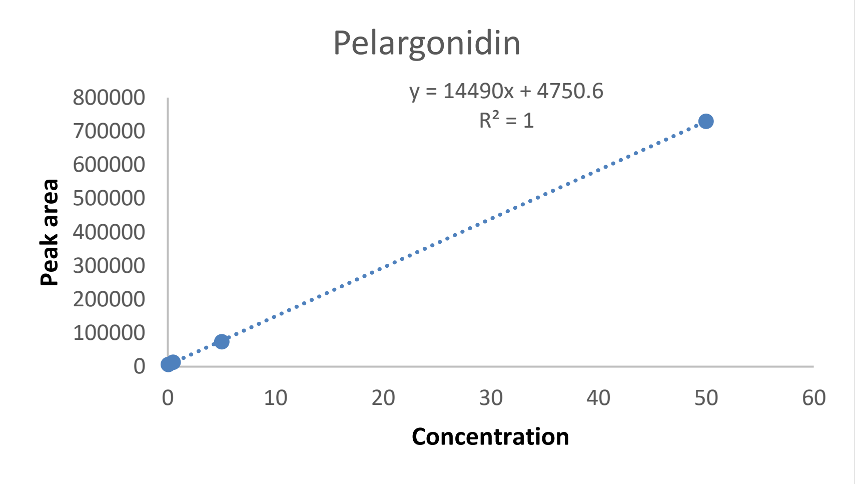
Pelargonidin
y = 14490x + 4750.6
R² = 1
800000
700000
600000
500000
400000
300000
200000
100000
0
0
10
20
30
40
50
60
Peak area
Concentration

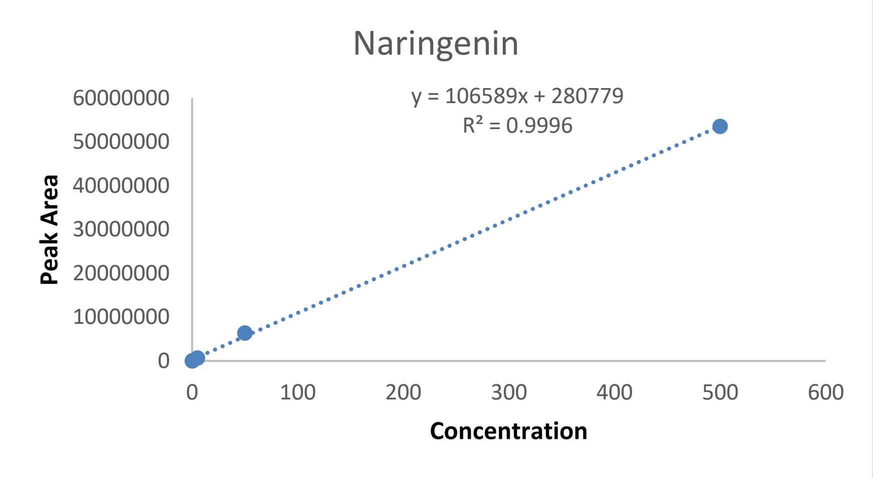
Naringenin
y = 106589x + 280779
R² = 0.9996
60000000
50000000
40000000
30000000
20000000
10000000
0
0
100
200
300
400
500
600
Peak Area
Concentration

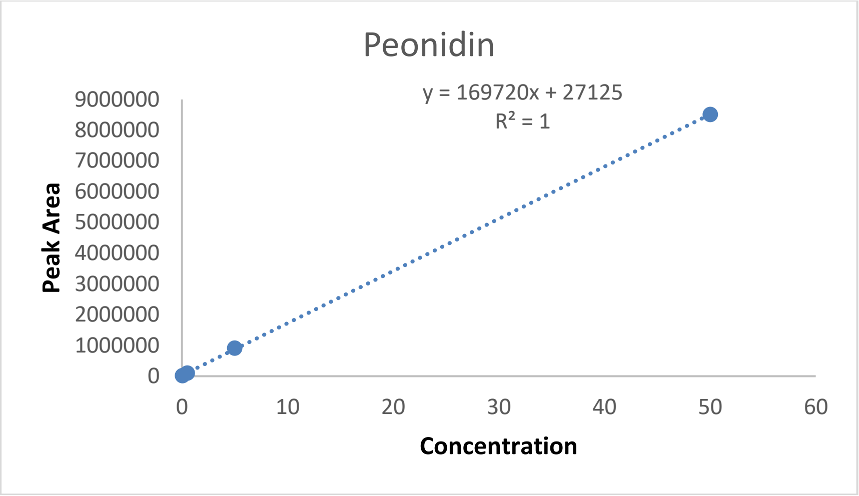
Peonidin
y = 169720x + 27125
R² = 1
Peak Area
9000000
8000000
7000000
6000000
5000000
4000000
3000000
2000000
1000000
0
0
10
20
30
40
50
60
Concentration

**Figure S2** –MS/MS chromatograms of different target flavonoids.

Naringenin-MS/MS

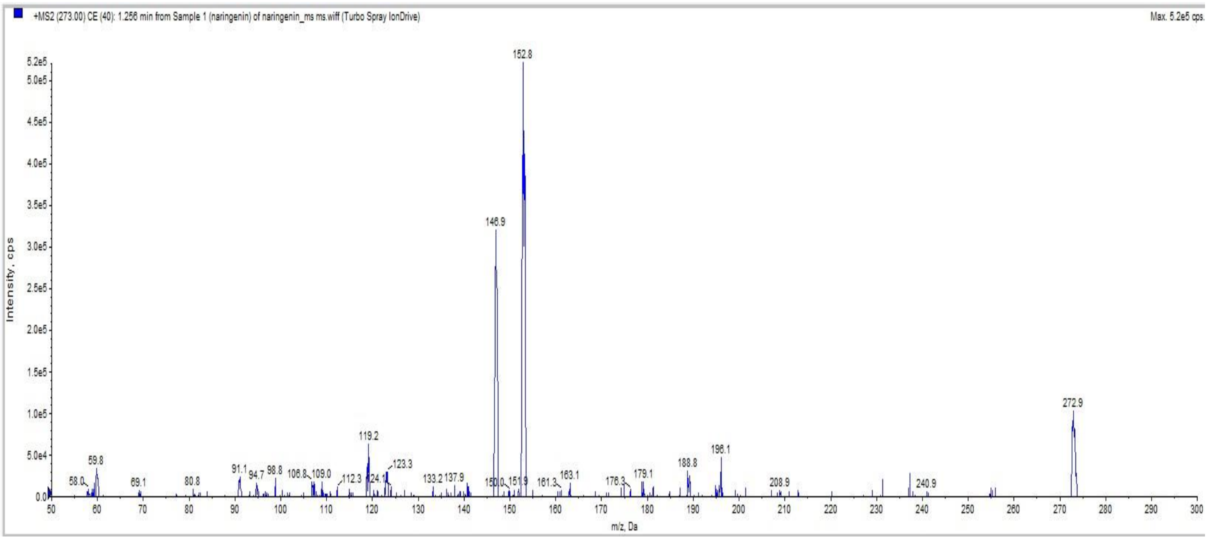


**Quercetin- MS/MS**

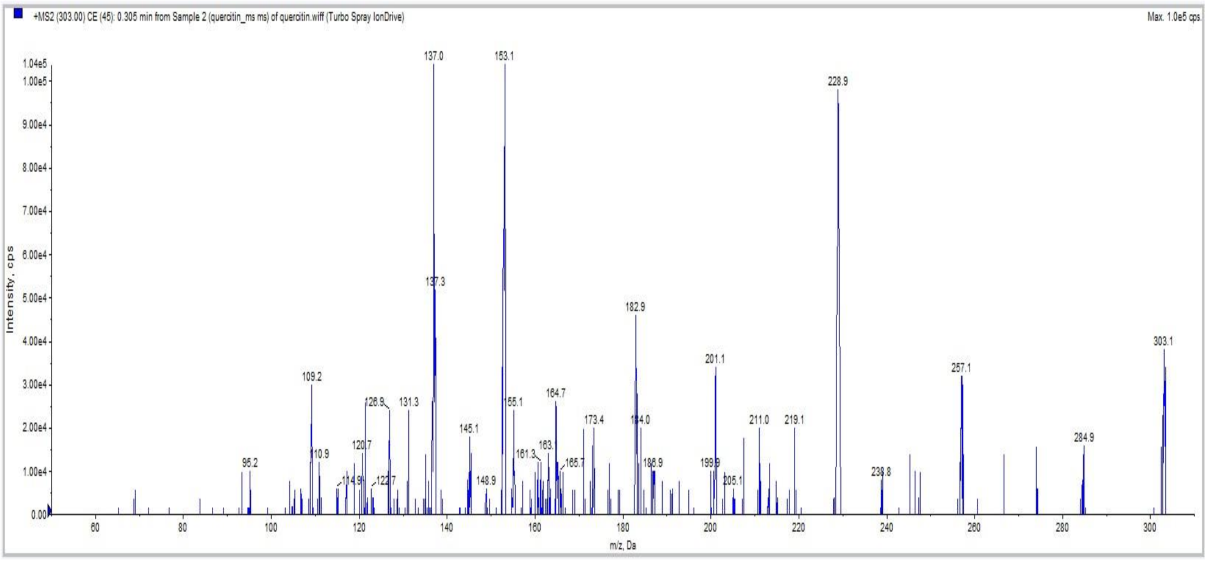


**Myricetin-MS/MS**

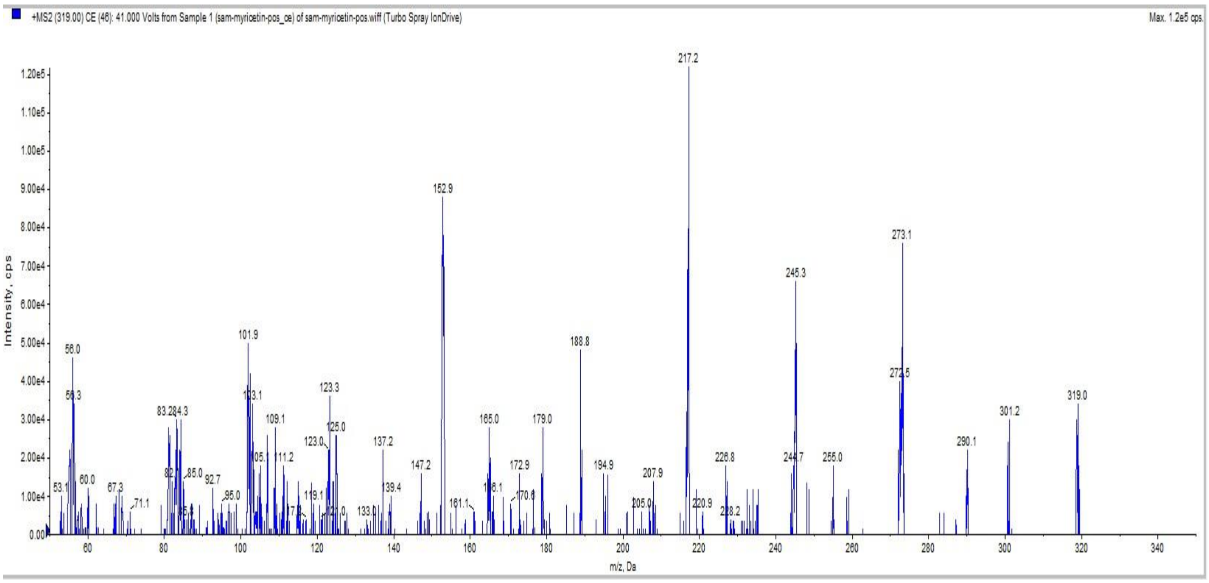


**Kaempferol-MS/MS**

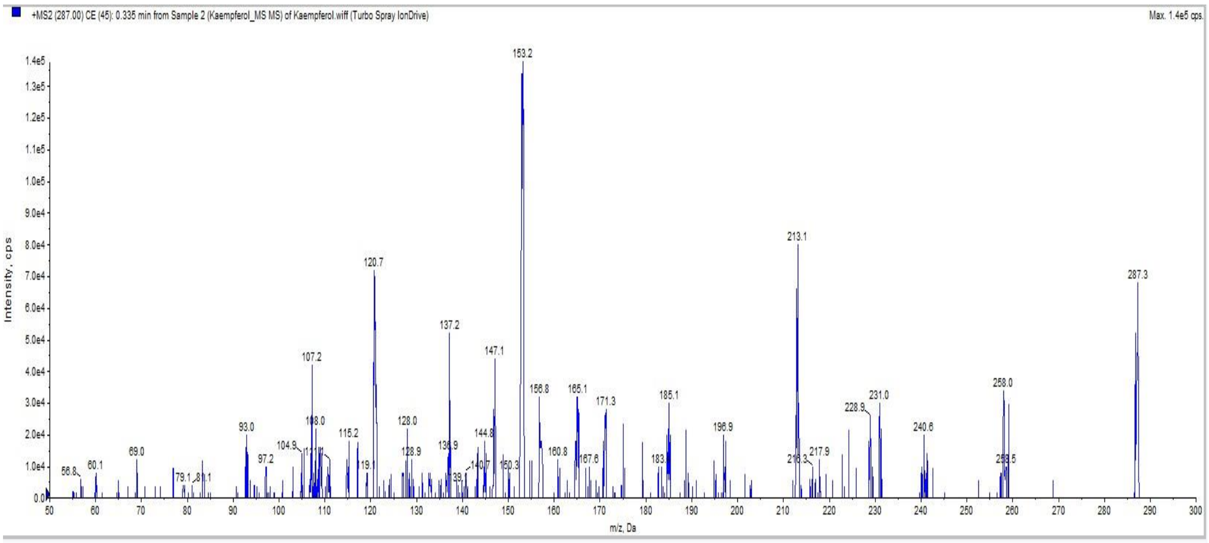


**Epigallocatechin-ms/ms**

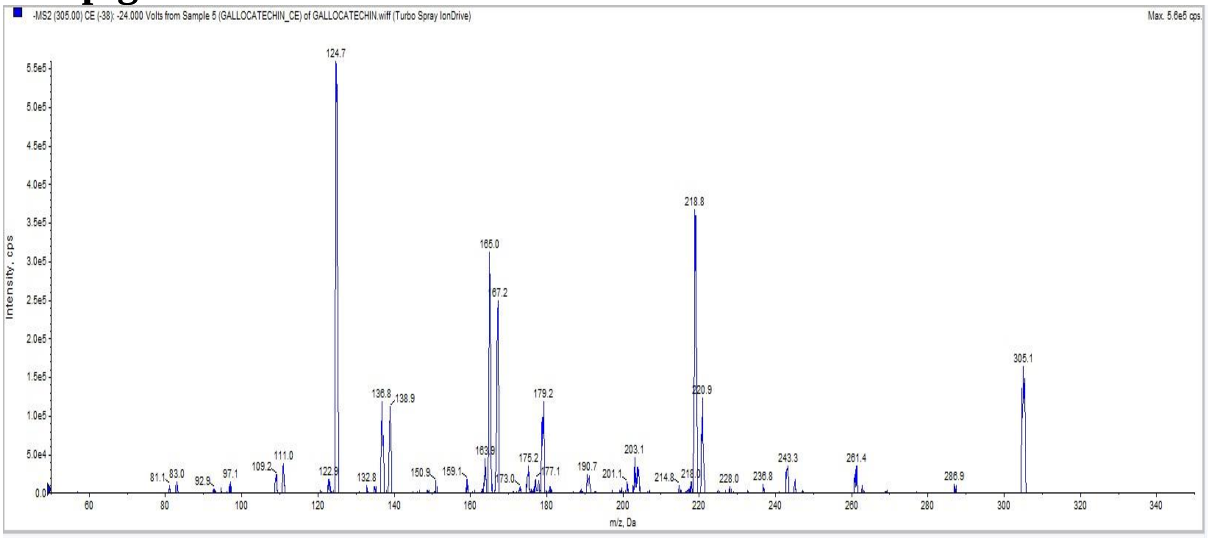


**Rutin-ms/ms**

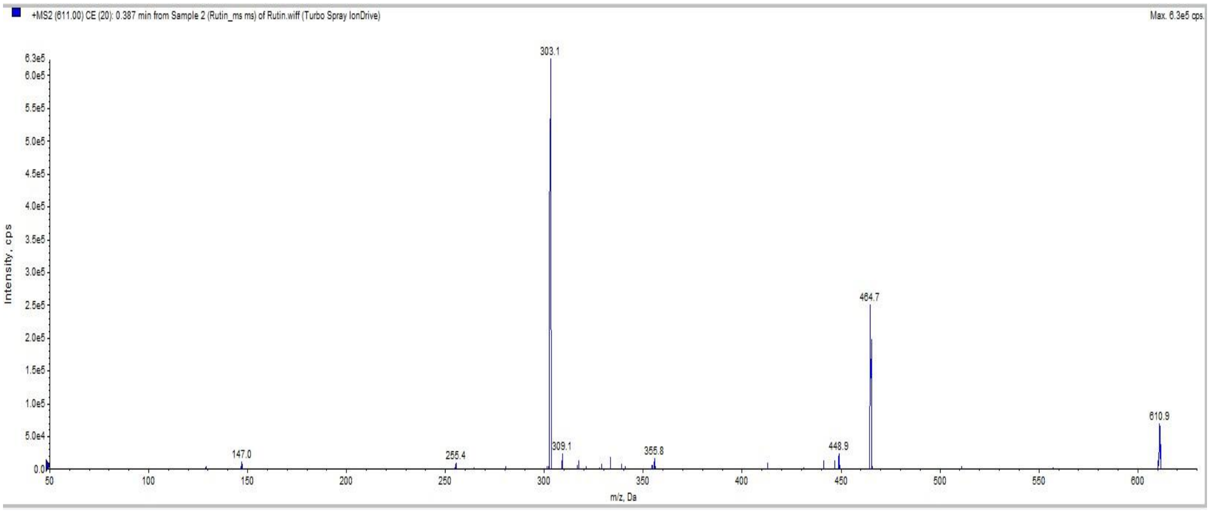


**Cyanidin-ms/ms**

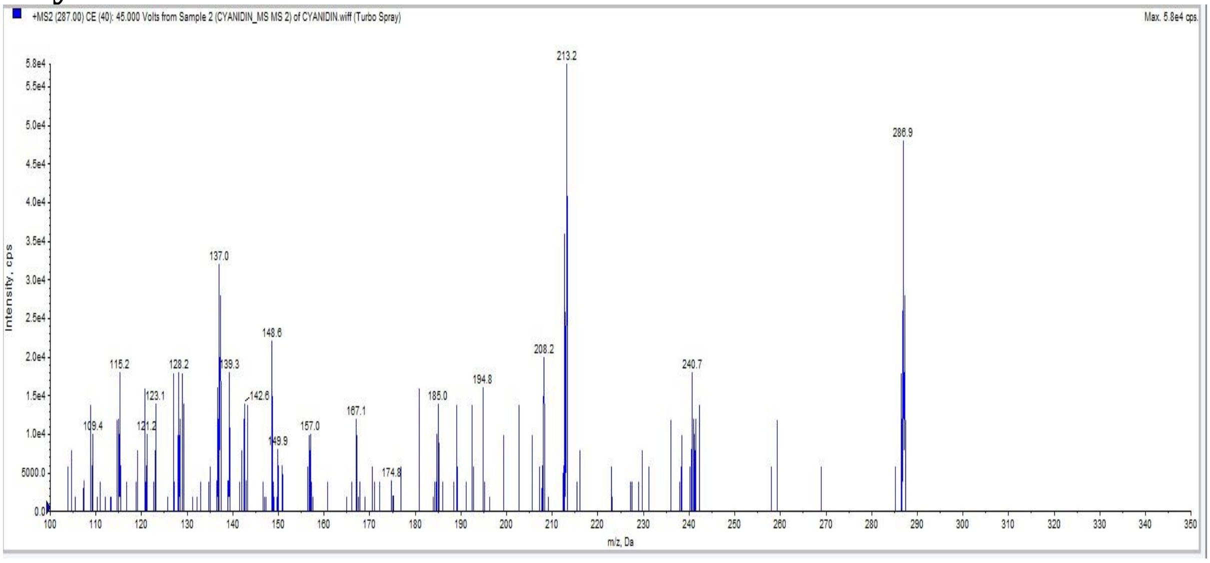


Pelargonidin-MS/MS

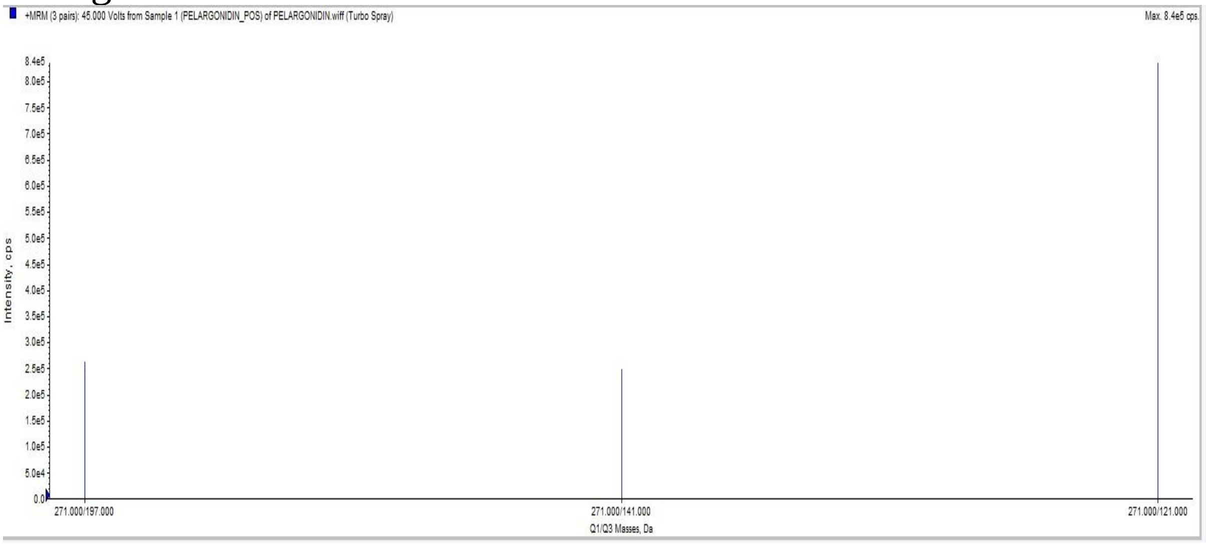


**Delphinidin- MS/MS**

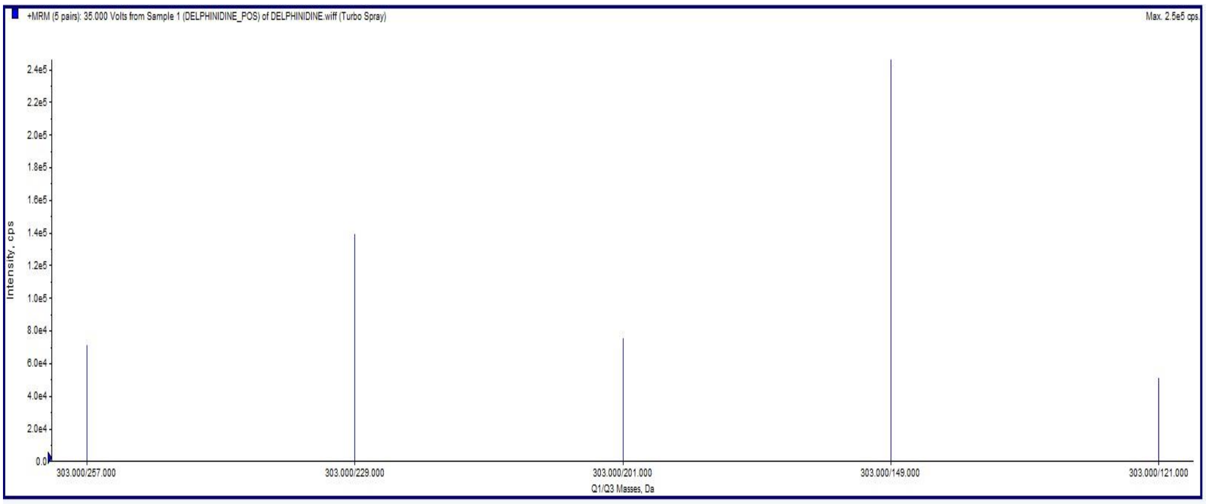


# Peonidin- MS/MS

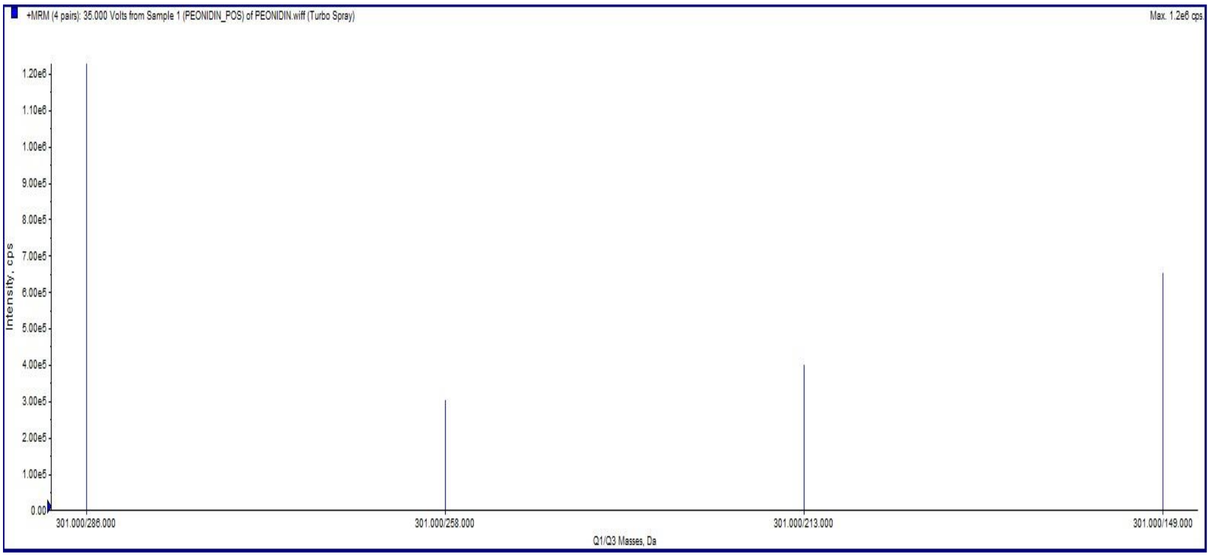

Supplement: Supplementary file 1 [file metabolites-14-00127-s001.zip › metabolites-2822902-supplementary.pdf]
